# Patterns of CDSS adoption in primary care: a cluster analysis and predictive modelling study from a stepped wedge trial

**DOI:** 10.1186/s13012-026-01516-0

**Published:** 2026-06-18

**Authors:** Jale Basten, Juliane Köberlein-Neu, Peter Ihle, Ingo Meyer, Nina Timmesfeld, the eHealth COMPATH Study Group

**Affiliations:** 1 Department of Medical Informatics, Biometry and Epidemiology, Ruhr University, Bochum, Germany; 2 Center for Health Economics and Health Services Research, Schumpeter School of Business and Economics, University of Wuppertal, Wuppertal, Germany; 3 PMV Research Group, Faculty of Medicine, University Hospital Cologne, University of Cologne, Cologne, Germany

**Keywords:** Adoption pattern, Clinical decision support system, eHealth solution, Hierarchical cluster analysis, Implementer engagement, Medication management, Predictive modelling, Primary care, Tailored implementation strategies

## Abstract

**Background:**

Despite the potential of eHealth solutions to enhance medication management and patient safety, the integration of clinical decision support systems (CDSSs) into primary care remains challenging for healthcare systems worldwide. In Germany, the Digital Healthcare Act created a legal framework to accelerate eHealth adoption, but implementation lags behind international standards. We reanalysed data from a completed cluster-randomised stepped wedge trial (SW-CRT) that implemented a CDSS for medication management in German primary care to identify and characterise distinct implementation patterns and their predictive factors.

**Methods:**

We linked routine health insurance records, practice structural data, pseudonymised CDSS logbook entries, and cross-sectional postal survey data from general practitioners (GPs) participating in the SW-CRT (*n* = 736 practices). We used hierarchical cluster analysis (Ward’s method) on five implementation outcomes to identify distinct adoption patterns. Random Forest models were developed to assess how well structural, patient-level, and attitudinal variables could classify practices into these patterns.

**Results:**

Of 736 participating practices, 356 (48%) performed at least one medication review. Hierarchical cluster analysis of these practices based on five implementation outcomes identified three distinct adoption patterns. The remaining 380 practices that did not perform medication reviews constituted a fourth pattern. CDSS usage intensity did not align with cluster-specific intervention effect across patterns. The pattern with the lowest usage intensity and fidelity showed the largest cluster-specific intervention effect on the combined endpoint of hospitalisation and mortality. Practices in this pattern reported significantly higher change commitment, change efficacy, and cognitive participation. Random Forest models using structural variables alone showed limited discrimination (AUC 0.56–0.66). Including a binary indicator of GP survey participation improved discrimination (AUC 0.61–0.82).

**Conclusions:**

Within this single trial context, higher CDSS usage intensity did not correspond to larger cluster-specific intervention effects, and adoption behaviour was heterogeneous across practices. Structural variables alone were insufficient to distinguish adoption patterns; differences were instead associated with attitudinal factors such as change commitment, change efficacy, and willingness to engage with the intervention. Because these attitudinal measures were collected after practices had reached intervention status, they cannot be interpreted as antecedents of adoption. These findings nonetheless underscore the value of assessing implementer engagement during implementation and of tailoring implementation strategies to distinct adoption patterns rather than pursuing uniform approaches.

**Trial registration:**

AdAM: ClinicalTrials.gov (NCT03430336), 6 February 2018; eHealth COMPATH: Open Science Framework (osf.io/gau5w), 29 December 2023.

**Supplementary Information:**

The online version contains supplementary material available at 10.1186/s13012-026-01516-0.

Contribution of literature • **Empirical Typology of Adoption Patterns**: We identified distinct CDSS adoption patterns among primary care practices, highlighting diverse needs and the importance of tailored implementation strategies.

• **Usage Intensity and Intervention Effects**: Within a single trial context, higher CDSS usage intensity and fidelity did not correspond to larger cluster-specific intervention effects, suggesting that intensity of use should not be equated with implementation success.

• **Limited Predictive Power of Structural Variables**: Our analyses showed that routinely available structural variables alone poorly distinguish CDSS adoption patterns, pointing instead to the role of implementer commitment and engagement during the adoption process.

## Background

The integration of clinical decision support systems (CDSS) into primary care remains a persistent challenge across healthcare systems worldwide [1]. Despite growing evidence for the potential of eHealth solutions to improve medication management and patient safety [2, 3], adoption rates remain low and implementation processes are often protracted [4]. In Germany, the Digital Healthcare Act (Digitale-Versorgung-Gesetz, 2019) created a legal framework to accelerate eHealth adoption, yet implementation continues to lag behind international standards [5–7]. Many literature reviews have identified and analysed barriers and facilitators contributing to this delay [8–18]. These reviews provided valuable catalogues of determinants, ranging from alert fatigue and workflow incompatibility to limited technology acceptance and organisational resistance. However, they predominantly analyse determinants at the aggregate level, treating adopters and non-adopters as homogeneous groups. How adopters and non-adopters differ in their implementation outcomes, and how far such differences can be identified using routinely available data, has received little attention. Understanding this heterogeneity is a prerequisite for designing implementation strategies that are tailored to the specific needs and capacities of different practice types, rather than pursuing uniform approaches.

This study addresses this gap through a secondary analysis of data from the AdAM project (Anwendung für ein digital gestütztes Arzneimitteltherapie- und Versorgungsmanagement or “application of digitally supported drug therapy and care management“; Federal Joint Committee Innovation Fund, grant: 01NVF16006). The AdAM project implemented a CDSS for medication management in primary care practices in the Westphalia-Lippe region of Germany. Each year, patients’ medication lists were reviewed using the CDSS. The CDSS generates warnings against inappropriate prescriptions based on health insurance data related to the patient. The AdAM study’s primary objective was to evaluate whether this CDSS could reduce hospitalisation and mortality in adult patients with polypharmacy [19, 20]. The trial did not demonstrate a statistically significant effect on this primary endpoint [20], and a separate quantitative process evaluation has examined the extent and quality of CDSS use across the trial [21]. The present secondary analysis differs in aim from both. Rather than estimating the overall intervention effect or describing aggregate process measures, it asks whether participating practices fall into distinct adoption patterns and how far these patterns can be characterised and distinguished using routinely available data. We draw on the implementation outcomes framework proposed by Proctor et al. (2011) [22] to operationalise and categorise adoption behaviours across participating practices.

We address these questions through two objectives: (1) To identify distinct adoption patterns among primary care practices participating in the AdAM trial based on implementation outcomes, and to characterise these patterns with respect to practice characteristics and attitudinal measures. (2) To develop and evaluate predictive models for classifying practices into these adoption patterns using a comprehensive set of variables, including patient characteristics, practice attributes, CDSS usage, attitudes toward polypharmacy, organisational readiness, software integration measures, project evaluation aspects, physicians’ beliefs about eHealth solutions, structural factors, and external influences.

## Methods

### Study design

This study is a secondary analysis of data from the AdAM trial, a pragmatic, cluster-randomised stepped wedge trial (SW-CRT) with an open cohort and a step length of one quarter (three months), conducted in primary care practices in the Westphalia-Lippe region of Germany from June 2017 to March 2021 [19, 20]. The analytical approach comprised two stages: (1) hierarchical cluster analysis to identify distinct adoption patterns, and (2) Random Forest modelling to evaluate the predictive value of available variables for pattern classification.

### Data source

We used data from various AdAM study work packages. Specifically, we used routine data from BARMER, a major German health insurance company, along with structural data from AdAM’s primary care practices. These data were analysed as part of the effectiveness evaluation [20]. We also used pseudonymised logbook entries of the CDSS previously evaluated in a quantitative process evaluation [21, 23], and data from a cross-sectional postal survey of AdAM’s general practitioners (GPs). The survey data were collected from September to December 2020 and assessed through a qualitative comparative analysis [24].

The scientific data warehouse (W-DWH) of the health insurance company BARMER served as a secondary data source. It contains fully anonymised routine data of BARMER-insured individuals and has no access to original identifiers. For the analyses, the original identifiers, such as the operating site number (*Betriebsstättennummer*, BSNR) and the lifetime physician number (*Lebenslange Arztnummer*, LANR) of the participating primary care practices and GPs, and those of the insured individuals recruited at the AdAM primary care practices, were pseudonymised and imported into the W-DWH. Analyses were conducted in the anonymised W-DWH database, with data linked at the individual level based on the identifying characteristics of the primary care practices (pseudonyms from BSNR). A trust center was established for this purpose. This linking allows for the use of various explanatory variables at both the individual and organisational levels to explain differences in implementation success.

This linking procedure was also employed to integrate structural data from primary care practices and AdAM GPs (provided by the regional Association of Statutory Health Insurance Physicians, the Kassenärztliche Vereinigung Westfalen-Lippe (KVWL)), pseudonymised logbook entries from RpDoc^®^ Solutions GmbH software utilised in AdAM (developed under the name “eMMa”, an abbreviation for “electronic medication management”), and postal survey data supplied by the Center for Health Economics and Health Services Research at the University of Wuppertal with the routine data stored in the W-DWH.

The survey [24] was designed specifically for the AdAM study and incorporated both self-designed items and validated measures, such as the Practice Adaptive Reserve (PAR) measure [25], the Organisational Readiness for Implementing Change (ORIC) measure [26], and the German Normalisation Process Theory (G-NoMAD) measure [27]. We assessed the internal consistency of each multi-item scale entering the analyses (ORIC Change Commitment and Change Efficacy; the G-NoMAD subscales; and the Practice Adaptive Reserve measure) using Cronbach’s α. The coefficients are reported in the supplementary material (Table S3). For data protection purposes, the KVWL distributed questionnaires to GPs actively participating in the project. At the time of distribution, the SW-CRT had been completed, and all primary care practices had reached intervention status. The attitudinal measures therefore represent cross-sectional assessments obtained after implementation rather than baseline dispositions. Following Dillmann’s approach [28], the KVWL sent two written reminders at two-week intervals. Physicians rated the items mostly on a 5-point Likert scale, where 1 indicated ‘strongly disagree’, 3 indicated ‘neither agree nor disagree’, and 5 indicated ‘strongly agree’.

### Implementers: primary care practices and GPs

In the context of the AdAM study, primary care practices in Westphalia-Lippe were informed about the study through regional media and contacted by the KVWL to invite participation. Additionally, the health insurance company BARMER sent informational flyers to its insured members in the region. Participating primary care practices were required to meet the following criteria:

- Provide health services for BARMER-insured patients,
- Employ physicians who are specialised in general medicine, internal medicine, or who do not have a designated speciality,
- Have at least ten potentially eligible patients (defined below),
- Have access to the KVWL website via a secure connection, and
- Obtain physician consent to fulfil the contractual obligations arising from the study.

Potentially eligible patients were defined as adult BARMER-insured individuals from the participating primary care practices who had at least five different medication prescriptions (defined by codes of the Anatomical Therapeutic Chemical (ATC) Classification documented in their routine health insurance records) over a minimum of two quarters. These patients were identified during the observation period using routine health insurance records.

### AdAM eHealth intervention

The experimental intervention of the AdAM study required participating GPs to use a CDSS in the treatment of their patients. The process has been described in more detail elsewhere [19, 20]. Briefly, GPs accessed the system via a secure web-based portal, receiving information at both the primary care practice level and, following patient consent, the individual patient level. The CDSS contained routine health insurance records, including patients’ medical histories, medication dispensed by pharmacies and co-treating physicians (with documented diagnoses and treatments), previous hospital treatments, and the use of remedies and aids. GPs entered patient-specific information (e.g., weight and kidney function) and confirmed or updated medication details (brand name, application route, and dosage). The CDSS assessed the medication and generated alerts in several categories, such as interactions, duplicate medications, and potentially inappropriate medication, assigning one of four severity levels [21, 23]. GPs could use the CDSS in the patient’s absence, optimise medication in response to alerts, and discuss treatment changes with the patient (Figure S1). Prior to the trial, GPs were invited to participate in intervention training, had access to the CDSS and a support hotline throughout the study, and received an annual reimbursement of €85 per patient for medication reviews.

GPs in the control condition had no access to the CDSS, and GPs in control or intervention conditions continued to provide standard care to their patients. According to the polypharmacy guideline for primary care [29], this should include a medication plan and an annual medication review.

### Statistical analysis

Our analysis is structured around two primary objectives. Firstly, we determine implementation outcomes to identify distinct adoption patterns among primary care practices participating in the AdAM trial. These outcomes align with the AdAM study’s endpoints and performance indicators and are categorised according to the “taxonomy” proposed by Procter et al. (2011) [22]. They include the cluster-specific intervention effect for the combined endpoint (the practice-level random intercept for the treatment effect on hospitalisation and mortality, hereafter the cluster-specific intervention effect), the time it takes primary care practices to adopt the CDSS (early versus late adoption), the proportion of enrolled patients relative to the potentially eligible patients (penetration), the proportion of enrolled patients who have had a medication review (fidelity), and the average number of CDSS actions performed per patient (intensity). By design, these outcomes are restricted to observable, behaviourally and administratively measurable dimensions of implementation. Perception-based implementation outcomes such as acceptability were not included. Related attitudinal constructs were instead examined separately as candidate explanatory variables. The operationalisation of these outcomes is detailed in Table 1.

**Table 1:** Operationalisation of the implementation outcomes

Outcome	Description
1. Cluster-specific intervention effect for the combined endpoint from the main analysis / sensitivity analysis of the AdAM study	The SW-CRT evaluated the impact of a user-initiated CDSS on the combined endpoint of hospital admissions and mortality in adults with polypharmacy. To capture variation in intervention effects across participating primary care practices, we calculated the practice-level random intercept for the treatment effect on hospitalisation and mortality derived from the primary AdAM effectiveness analysis. Primary care practices (clusters) were excluded from the main analysis if they had no patients participated during the intervention period. Therefore, the cluster-specific intervention effect applies only to primary care practices that enrolled at least one patient. The AdAM analysis model was based on a generalised linear mixed model including fixed effects for time (quarter), treatment group, sex, age, medication-related chronic disease prognostic score (medCDS) [30], and level of nursing care. To account for clustering and repeated measures, the model includes one uncorrelated random patient effect and two correlated random cluster level effects. As a sensitivity analysis, we recalculated the main analysis model including all primary care practices, regardless of patient enrolment status. This analysis was restricted to the pre-pandemic period, up to March 31, 2020. In the present study, cluster-specific intervention effects derived from the main analysis were used whenever available. For practices without an available estimate from the main analysis - due to the absence of patient enrolment under the corresponding operating site number (BSNR) - the corresponding estimate from the sensitivity analysis was used instead.
2. Time from the start of primary care practice participation to the first application of the CDSS (early versus late adoption)	For each primary care practice, we calculated the time (in days) from the activation date in the KVWL portal, after the GP declared participation, to the first use of the CDSS. To determine this variable, we selected the earliest date on which an AdAM GP in the primary care practice met one of the following criteria (eMMa action criteria): 1. Confirmation by the GP in the software that a patient history (anamnesis) had been completed (referred to as ‘completed anamnesis’). 2. Use of the software to print a medication plan. 3. Entry of at least five medications into the software.
3. Proportion of enrolled patients to potentially eligible patients (penetration)	The proportion of enrolled patients to potentially eligible patients is calculated by dividing the number of patients who provided written informed consent to participate in the study and were treated by an AdAM GP during the intervention phase (numerator) by the number of patients from the primary care practice who met the inclusion criteria during the same phase (denominator).
4. Proportion of enrolled patients who have had a medication review (fidelity)	A patient was considered to have received a medication review at the primary care practice if they met at least one of the three eMMa action criteria (see implementation outcome 2). The denominator for our analysis included the number of consenting AdAM study participants who received care during the practice’s intervention phase.
5. Average number of CDSS actions performed per patient prescribed in the AdAM study and treated in the corresponding primary care practice (intensity)	The number of CDSS actions performed per patient prescribed in the AdAM study and treated in the corresponding primary care practice, where an action is defined as meeting at least one of the three eMMa action criteria (see implementation outcome 2).

We employed hierarchical cluster analysis to identify distinct adoption patterns. For the hierarchical cluster analysis, we excluded primary care practices from the sample that did not perform medication reviews because they represented a distinct adoption pattern. Because the implementation outcomes differed in scale and measurement units, all implementation outcomes were standardised prior to analysis to ensure equal variable contribution.

To determine the optimal clustering solution, we visually inspected the resulting dendrograms and evaluated clustering performance using the Silhouette index. To assess the robustness of the clustering solution, analyses using different agglomeration methods (Ward, complete, and average agglomeration) and distance metrics (Euclidean and Manhattan distance) were conducted. The final clustering specification was selected based on statistical performance, interpretability, and clinically meaningful group distributions.

Differences in implementation outcomes across adoption patterns were assessed using one-way analysis of variance (ANOVA) for normally distributed variables (cluster-specific intervention effects) and Kruskal-Wallis test for non-normally distributed continuous variables.

Descriptive comparisons of practice characteristics, survey measures, and candidate explanatory variables across adoption patterns were performed using non-parametric Kruskal-Wallis tests for continuous variables and chi-squared tests for categorical variables. Continuous variables are presented as medians with interquartile ranges (IQR). Reported p-values refer to overall comparisons across all identified adoption patterns.

Secondly, we developed and evaluated predictive models using advanced machine learning techniques, specifically Random Forest (RF) models (implemented using the *ranger* package in R [31]), for prospective classification of practices into adoption patterns. The model incorporated various factors, such as patient characteristics, practice attributes, AdAM software usage, attitudes toward polypharmacy, organisational readiness, software integration measures, project evaluation aspects, physicians’ beliefs about eHealth solutions, structural factors, and external influences. Random Forest is an ensemble machine learning method that aggregates a large number of decision trees, each grown on a bootstrap sample of the data and a random subset of predictors and combines their outputs by majority vote. Its properties are well suited to our data. Specifically, it accommodates continuous and categorical predictors simultaneously, captures non-linear relationships and interactions without requiring them to be pre-specified, is comparatively robust to correlated predictors and to a large number of candidate variables relative to the number of practices, makes no strong distributional assumptions, and yields measures of variable importance. To explore the robustness of the predictive findings across different analytical approaches, Gradient Boosting models (implemented using the *gbm* package in R [32]) were additionally evaluated.

We divided the dataset into a training set (two-thirds) and a testing set (one-third). The RF model was fitted using the training set. We evaluated the model’s performance on the test set and assessed its predictive accuracy using Receiver Operating Characteristic (ROC) curves and the corresponding Area Under the Curve (AUC) values to compare its discriminative ability. All statistical analyses were performed using R version 4.3.1.

## Results

From June 2017 to July 2019, 1,348 primary care practices in Westphalia-Lippe were invited to participate in the AdAM study. These practices were identified by their main operating site number (*Hauptbetriebsstättennummer*, HBSNR). A total of 937 GPs agreed to participate. However, 16 AdAM GPs were excluded from the analysis because they had no eligible patients, withdrew from the study, or died during the study period. Since software access was linked to individual GPs, multiple physicians from the same operating site could participate. Additionally, GPs could change operating sites or work across multiple (main/secondary) operating sites during the study. Consequently, the final analysis sample included a total of 736 participating primary care practices.

**Fig. 1:**
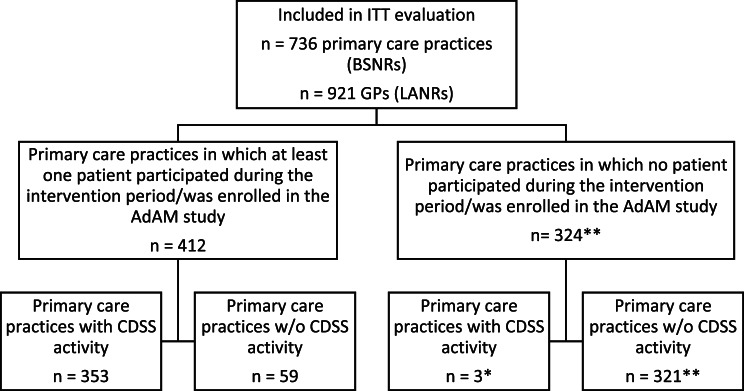
Flow chart. * Since access to the software was linked to the GPs, the randomisation of the main operating sites (HBSNRs) determined the GPs’ group allocation. During the study, a total of 64 GPs changed primary care practices or worked in several primary care practices simultaneously. This resulted in more clusters (primary care practices) than randomised main operating sites (HBSNR) where the AdAM GPs worked. Therefore, the GP could enrol their patient under a different operating site number (BSNR) than the one in which they used the CDSS software for that patient. ** Twelve primary care practices had no potentially eligible patients during their intervention phase. These practices were excluded from the hierarchical cluster analysis, as they could not voluntarily decide for or against the adoption of the CDSS

Of the participating primary care practices, 44% (324/736) did not enrol any patients during the intervention period. Of the remaining 412 primary care practices, 14% (59/412) did not perform a medication review using the CDSS. Notably, three primary care practices without enrolled patients still conducted a medication review for at least one patient. This occurred because GPs were given access to the software to use it for their enrolled patients across different operating sites (BSNR). Overall, at least one medication review was conducted in 48% (356/736) of all participating primary care practices (Fig. 1), while no medication review was performed in the remaining 52% (380/736).

### Classification of adoption patterns

Before conducting the hierarchical cluster analysis, the dataset was adjusted by excluding the 380 primary care practices (52%) that did not perform a medication review. This is because these practices represent a distinct adoption pattern, referred to as Pattern 4 and described in more detail later. Twelve primary care practices of this pattern had no potentially eligible patients undergoing treatment during the intervention phase and were therefore unable to perform a medication review. The hierarchical cluster analysis included 356 primary care practices (48%). In all cases where the cluster-specific intervention effect from the main analysis model was available (*n* = 353), only that effect was included in the hierarchical cluster analysis. For the remaining three primary care practices where the main analysis model’s effect was unavailable, the cluster-specific intervention effect from the sensitivity analysis was used.

Different combinations of distance metrics and agglomeration methods were evaluated to identify a robust and interpretable clustering solution (Table S4). Among the examined specifications, hierarchical clustering using Euclidean distance and Ward’s agglomeration method identified three adoption patterns among practices performing medication reviews and provided the best balance between interpretability, clinically meaningful group distributions, and cluster separation. Although some clustering specifications resulted in higher Silhouette indices, these solutions produced highly imbalanced group sizes with limited practical interpretability.

**Fig. 2:**
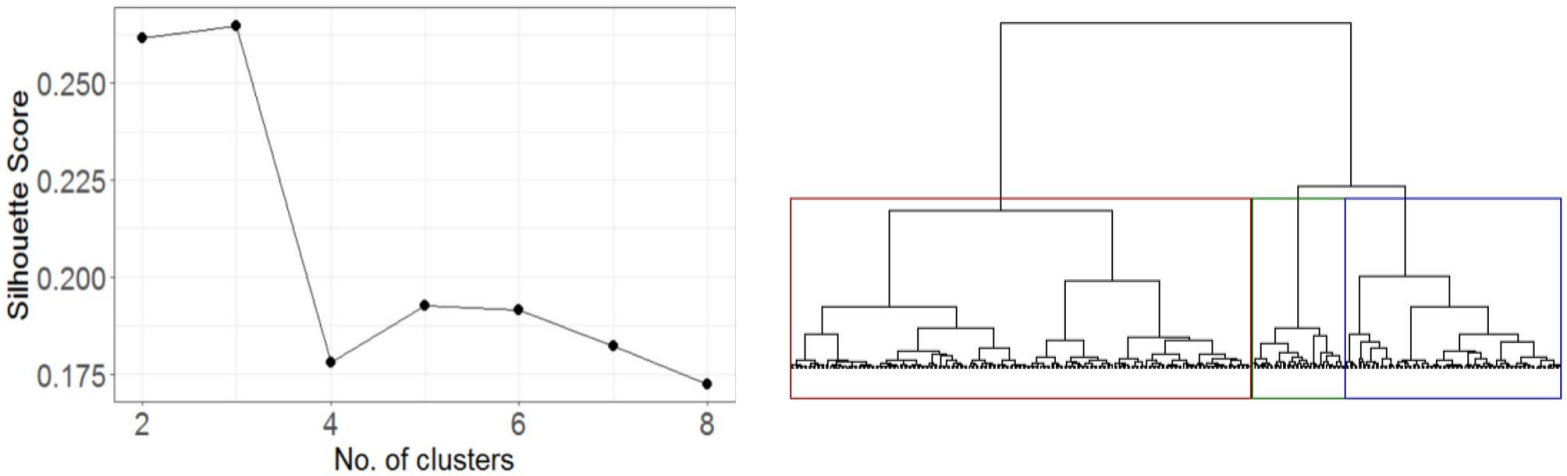
Silhouette index and dendrogram of the hierarchical cluster analysis

The Silhouette index was calculated to evaluate the clustering solution. The highest mean Silhouette index (0.265) was observed for the three-pattern solution (see Fig. 2). Including the adoption pattern of primary care practices without medication review yields a four-pattern solution. This four-pattern solution was therefore selected for further analysis. The characteristics of the resulting adoption patterns are as follows:

- **Pattern 1 (*n*** **= 43**,** 6%)**: Primary care practices with this adoption pattern demonstrated the greatest cluster-specific intervention effects (mean = -0.025, SD = 0.092). The median time from activation to the first use of the CDSS was 117 days (IQR: 46–219). A median of 21% (IQR: 10%-40%) of potentially eligible patients were enrolled, with a median of 47 (IQR: 34–65) potentially eligible patients per practice. Among those enrolled, 30% (IQR: 20%-50%) received a medication review. Furthermore, the median number of CDSS activities per patient was 0.87 (IQR: 0.2–1.4), indicating relatively low engagement with the CDSS platform.
- **Pattern 2 (*n*** **= 213**,** 29%)**: Primary care practices with this adoption pattern exhibited the second-highest cluster-specific intervention effects (mean = -0.013, SD = 0.076). The median time from activation to the first use of the CDSS was 61 days (IQR: 22–116). In median, 57% (IQR: 50%-70%) of potentially eligible patients were enrolled, with a median of 52 (IQR: 35–77) potentially eligible patients per practice. Among those enrolled, 98% (IQR: 90%-100%) received a medication review. The median number of CDSS activities per patient was 5.34 (IQR: 4.2–6.5), indicating a high level of engagement with the CDSS platform.
- **Pattern 3 (*n*** **= 100**,** 14%)**: Primary care practices with this adoption pattern exhibited the lowest cluster-specific intervention effects (mean = 0.022, SD = 0.080). The median time from activation to the first use of the CDSS was 58.5 days (IQR: 9.8–110). In median, 19% (IQR: 10%-30%) of potentially eligible patients were enrolled, with a median of 67 (IQR: 46–94) potential patients per practice. Among those enrolled, 100% (IQR: 90%-100%) received a medication review. The median number of CDSS activities per patient was 1.88 (IQR: 0.8–2.7).
- **Pattern 4 (*n*** **= 368**,** 51%)**: For this pattern, the cluster-specific intervention effect was taken from the sensitivity-analysis model, because most of these practices did not enroll patients and were therefore not represented in the main-analysis model. In this model, the effect for Pattern 4 lay between those of Patterns 2 and 3. In median, primary care practices with this adoption pattern had 35 (IQR: 21–53) potentially eligible patients.

Table 2 summarises the mean or median implementation outcomes for each of the four adoption patterns. For Patterns 1 to 3, the cluster-specific intervention effect is taken from the main-analysis model, which is available for practices that enrolled at least one patient. For Pattern 4, which consists predominantly of non-enrolling practices, the effect is taken from the sensitivity-analysis model, which included all practices regardless of enrolment and was restricted to the pre-pandemic period. Both analyses are reported in Table 2 for comparability.

**Table 2:** Characteristics of adoption patterns derived from hierarchical cluster analysis

Implementation outcome	All (*n* = 724)	Pattern 1 (*n* = 43)	Pattern 2 (*n* = 213)	Pattern 3 (*n* = 100)	Pattern 4 (*n* = 368)	*p*-value*
Cluster-specific intervention effect (main analysis)	0.003 (0.079)	-0.025 (0.092)	-0.013 (0.080)	0.022 (0.080)	0.004 (0.069)	0.001
Cluster-specific intervention effect (sensitivity analysis)	-0.032 (0.022)	-0.032 (0.023)	-0.034 (0.024)	-0.026 (0.023)	-0.032 (0.019)	0.024
Time from start to first CDSS use in days (early versus late adoption)	63.5 [20; 124]	117 [46; 219]	61 [22; 116]	58.5 [9.8; 110]	Inf [Inf; Inf]	0.006
Prop. of enrolled patients (penetration)	0.04 [0; 0.5]	0.21 [0.1; 0.4]	0.57 [0.5; 0.7]	0.19 [0.1; 0.3]	0 [0; 0]	< 0.001
Prop. of enrolled patients with medication review (fidelity)	0.96 [0.6; 1]	0.3 [0.2; 0.5]	0.98 [0.9; 1]	1 [0.9; 1]	0 [0; 0]	< 0.001
No. of CDSS actions per patient (intensity)	3.95 [1.9; 5.8]	0.87 [0.2; 1.4]	5.34 [4.2; 6.5]	1.88 [0.8; 2.7]	0 [0; 0]	< 0.001

* P-values refer to overall comparisons across adoption patterns using one-way ANOVA for cluster-specific intervention effects and Kruskal-Wallis tests for all other implementation outcomes

The supplementary material (Figures S2-S7) provides boxplots showing the distribution of implementation outcomes for each adoption pattern.

Additional exploratory analyses of eMMa action criteria usage revealed differences across adoption patterns. Among practices meeting at least one of the three eMMa action criteria, Pattern 1 more frequently entered at least five medications into the software (34%) compared to Pattern 2 (22%) and Pattern 3 (25%). In contrast, practices in Patterns 2 and 3 more frequently confirmed completion of a patient history (anamnesis) (Pattern 1: 41%, Pattern 2: 54%, Pattern 3: 53%). These descriptive differences suggest variation in how practices interacted with the CDSS workflow across adoption patterns.

### Characteristics of adoption patterns

Table S2 shows the descriptive statistics for all possible predictive factors across the adoption patterns. Cronbach’s α coefficients indicated good to very good internal consistency for all questionnaire-based measures and subscales used in the study (α = 0.70–0.97; see Table S3). The four adoption patterns showed differences regarding practice characteristics, adaptability to change, and readiness to implement the AdAM software.

Patterns 2 and 4 consist primarily of single-handed practices, while Patterns 1 and 3 include a higher proportion of group practices. Panel sizes vary across patterns, with Pattern 3 having the largest panels on average. GPs in Patterns 1, 2, and 3 are younger on average than those in Pattern 4. Specialisation patterns differ, with Pattern 1 having a higher percentage of internists active in primary care, while the other patterns have a greater proportion of physicians specialised in general practice. Notably, Pattern 2 has a higher proportion of female GPs than the other patterns.

Participation in the postal survey regarding stakeholder experiences with implementing the CDSS differed substantially across adoption patterns. Response rates were lowest among Pattern 4 practices (38 out of 368, 10.3%) and highest among Pattern 2 practices (158 out of 213, 74.2%), followed by Pattern 3 practices (60 out of 100, 60%) and Pattern 1 practices (17 out of 43, 39.5%).

Survey results overall statistically significant differences across adoption patterns regarding organisational readiness for implementing change. Mean scores for Change Commitment (ORIC items 1–5) and Change Efficacy (ORIC items 6–9) were highest in Pattern 1 (Table S2). In accordance with recommendations from the authors of the German ORIC version [26], item 10 was excluded from the Change Efficacy subscale due to its poor performance in the pretest.

Similarly, mean Cognitive Participation scores (G-NoMAD Part B), reflecting self-reported willingness to participate in and collectively support the intervention, were highest among GPs in Pattern 1. No statistically significant differences across adoption patterns were observed for the remaining G-NoMAD Part B subscales (Coherence, Collective Action, and Reflexive Monitoring).

### Prediction of implementation behaviour

We assessed the ability of the RF models to classify practices into adoption patterns by comparing their ROC curves and corresponding AUC values. Low response rates for the postal survey posed challenges. We could either use a small amount of fully completed data from the surveyed practices or exclude the survey questions from the prediction model. Table S1 shows the proportion of possible predictive factors that were filled in across all adoption patterns (1–4) or Patterns 1–3.

**Fig. 3:**
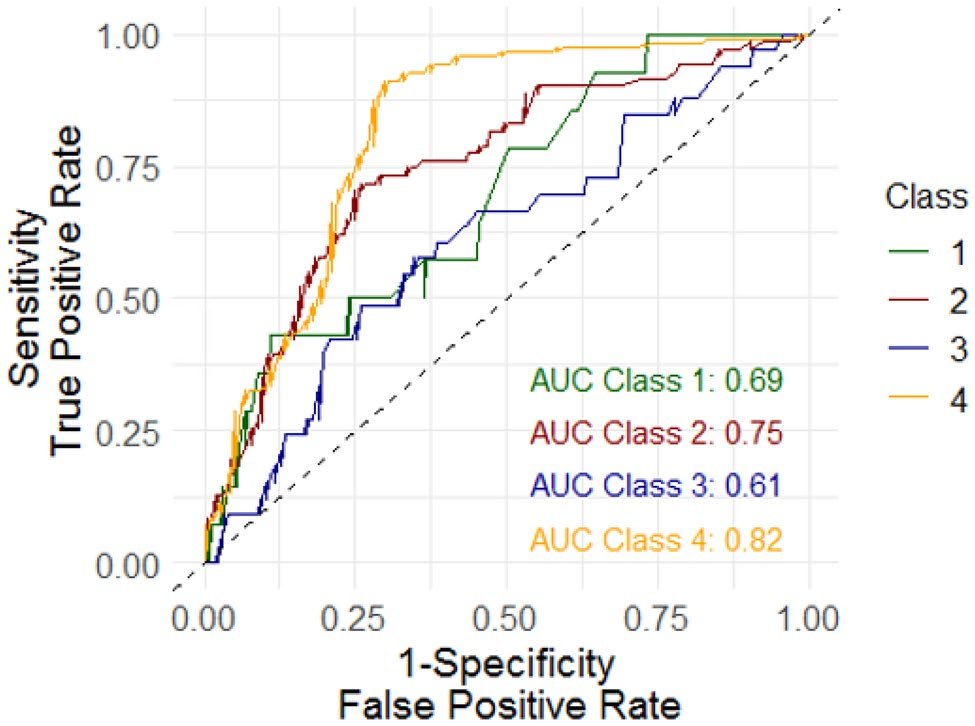
Receiver Operating Characteristic (ROC) curves and corresponding Area Under the Curve (AUC) values for the Random Forest (RF) model across four distinct adoption patterns. The RF model includes patient characteristics, practice profile information, randomisation wave, and an indicator that shows whether the GP participated in the survey

Across all patterns, the RF model incorporating patient characteristics (age, sex, medication-related chronic disease prognostic score (medCDS) [30], and level of nursing care), practice profile information (age and sex of GPs, physician specialisation, practice type, panel size, and GP network membership), and randomisation wave achieved AUC values ranging from 0.56 to 0.66 (Figure S8). Incorporating an indicator showing whether the GP participated in the survey improved classification performance, with AUC values ranging from 0.61 to 0.82. (see Fig. 3).

Including all or some of the survey questions did not improve the classification performance. This also reduces the available data because the RF model only considers complete data (see Figure S9 and S10).

Gradient Boosting demonstrated lower classification performance across adoption patterns and were therefore not pursued further (Figures S11-S14).

## Discussion

This study examined the heterogeneity of CDSS adoption behaviours within a single implementation context, which is an aspect that has received limited attention in the predominantly aggregate-level literature on barriers and facilitators of CDSS adoption [8–18]. Using hierarchical cluster analysis, we identified four distinct adoption patterns among 736 primary care practices participating in a stepped wedge trial of a CDSS for medication management (AdAM trial). Two findings are of particular relevance: (1) higher CDSS usage intensity was not consistently associated with stronger patient-level intervention effects, and (2) routinely available structural variables showed limited ability to distinguish between adoption patterns.

### Implementation outcomes and intervention effects

Pattern 1 showed the largest cluster-specific intervention effect despite the lowest usage intensity and fidelity among adopting practices. Only 30% of enrolled patients in Pattern 1 received a medication review, compared with nearly all patients in Patterns 2 and 3. This apparent dissociation between usage intensity and the cluster-specific intervention effect should be interpreted with caution, because Pattern 1 comprised only 43 practices with few enrolled and reviewed patients, so the underlying random intercepts are estimated with considerable uncertainty and the difference may partly reflect statistical noise or regression to the mean rather than a genuine advantage of selective use. With this caveat, the observation is consistent with Páez et al. (2025), who reported that high fidelity yields more accurate effectiveness assessments but does not necessarily increase effectiveness itself [33].

Furthermore, the relatively low proportion of enrolled patients receiving medication reviews may have limited the likelihood of detecting stronger patient-level effects on hospitalisation and mortality. Even when medication reviews were conducted, not all reviews necessarily resulted in clinically meaningful medication changes capable of influencing these outcomes.

Alternative explanations for the observed variation in cluster-specific intervention effects should also be considered. Practices with more favourable effects may differ in unmeasured organisational, motivational, or contextual characteristics that were not fully captured by the available structural and survey data.

The observed differences in eMMa action criteria usage suggest variation in how practices interacted with the CDSS workflow across adoption patterns. Pattern 1 more frequently focused on entering medication information, whereas Pattern 2 and 3 more often completed workflow-related steps such as confirming patient history documentation. These differences may reflect a more targeted and selective use of the CDSS in Pattern 1, focusing primarily on pharmacological alert content rather than completion of the full workflow as originally intended in the AdAM project.

The broader CDSS literature has identified alert fatigue as a major barrier to effective adoption [34–38]. Practices that process high volumes of alerts routinely may be more susceptible to alert fatigue than practices that engage selectively with the system. Additionally, the lack of integration between the AdAM software and practice information systems may have differentially affected the patterns. Practices with higher usage intensity may have experienced greater workflow disruption, reducing the effectiveness of the intervention despite high fidelity [34].

### Attitudinal differences across adoption patterns

The survey revealed that Pattern 1 practices demonstrated significantly higher change commitment, change efficacy (ORIC), and cognitive participation (G-NoMAD) compared to the other patterns. No statistically significant differences were observed for the remaining G-NoMAD Part B subscales Coherence, Collective Action, and Reflexive Monitoring. This profile suggests that adoption pattern with the highest cluster-specific intervention effect differed less in terms of understanding of the intervention or organisational capacity, and more in terms of motivational disposition, reflected in higher self-reported change commitment, confidence in managing the required tasks, and willingness to engage with the intervention. These findings extend the work of Söling et al. (2023) and Söling et al. (2020), who identified three factors relevant to physicians’ willingness to implement the AdAM intervention components, in particular their interpretation of the relevance of pharmaceutical knowledge provided by the CDSS, their medical code of ethics in the context of digitalisation, and their concepts of evidence-based medicine based on professional experience with polypharmacy [39, 40]. Our analysis adds a quantitative dimension to these qualitative findings by demonstrating that motivational and attitudinal constructs differed across adoption patterns that also differ in their patient-level outcomes.

However, the survey was administered at the end of the study, after all practices had reached intervention status. The observed attitudinal differences may therefore partly reflect retrospective evaluation of implementation experiences rather than baseline dispositions. Practices that successfully adopted the CDSS may report higher commitment and self-efficacy as a consequence of their implementation experience rather than as a precondition. This potential for reverse causality cannot be resolved with the available data and should be considered when interpreting the attitudinal findings.

### Predictive modelling

RF models incorporating structural variables, in particular patient characteristics, practice profile information, and randomisation wave, demonstrated only modest classification performance (AUC 0.56–0.66). These results suggest that routinely available administrative data alone may have limited ability to distinguish between adoption patterns. Including a binary indicator of GP survey participation improved discrimination (AUC 0.61–0.82). However, survey participation was not designed as a measure of commitment or engagement and should not be interpreted as a direct indicator of these constructs. The observed association may reflect multiple factors. In addition, its practical value for early identification of adoption patterns is limited, as it is unavailable before implementation begins.

The relatively low predictive performance may reflect the complexity of adoption behaviour, which is driven by motivational, attitudinal, and contextual factors that are not captured in administrative databases. For implementation planning, these findings suggest that implementer engagement and attitudinal readiness may be relevant considerations during implementation planning, as structural profiling alone is unlikely to suffice for identifying practices that require targeted support. In addition, the moderate cluster separation indicated by the Silhouette index suggests partial overlap between adoption patterns. This limited separability may have contributed to the modest predictive performance of the Random Forst models, as practices could not always be clearly assigned to distinct behavioural groups. More broadly, the limited gain from combining individually linked routine data with machine learning tempers expectations that such methods are, by themselves, sufficient to anticipate implementation behaviour, which is itself an informative result given the prominence these approaches have been afforded in recent methodological debates.

### Implications

Our findings have three practical implications. Firstly, implementation strategies should not assume that uniformity and high fidelity are optimal goals, particularly when fidelity is defined as the use of intervention components for every patient in the intervention group, without evaluating their appropriateness on an individual basis. Our data are compatible with selective, targeted use being as effective as comprehensive routine use, although this remains a hypothesis to be tested prospectively rather than a demonstrated effect. Implementation support might therefore accommodate different adoption styles rather than providing standardised workflows. Second, the assessment of change commitment and engagement should be integrated into implementation monitoring from the outset. This is especially relevant not only for post-hoc evaluation, but for the prospective identification of practices that may require additional support. Third, the heterogeneity of adoption patterns observed within a single trial context underscores the need for tailored rather than uniform implementation strategies. The reanalysis of the AdAM data was conducted as part of the eHealth COMPATH project [41]. This project aims to build on these findings by incorporating adoption pattern profiling into the design of context-specific implementation strategies.

### Limitations

This study offers insights into CDSS adoption patterns in primary care, but several limitations should be considered when interpreting the findings. First, this is a secondary analysis of data not originally collected for the current research questions, which constrains the available variables and their operationalisation. Nonetheless, it draws on a comprehensive dataset combining postal surveys, CDSS logbook entries, structural practice data, and routine health insurance records, which provides a broad foundation for the analyses. Second, survey response rates were low, particularly in Pattern 4 (10.3%). This introduces potential selection bias and means that the attitudinal measures, especially for non-adopting practices, may not be representative. The attitudinal differences we observed should therefore be regarded as provisional and would require confirmation in samples with more complete response. Given the highly differential survey response rates across adoption patterns, particularly among non-adopting practices, multiple imputation approaches were considered inappropriate because the missingness mechanism was unlikely to satisfy Missing At Random assumptions. Third, the survey was administered at the end of the intervention phase, so attitudinal measures may reflect retrospective evaluations of implementation experiences rather than baseline dispositions. This precludes a causal interpretation. Prospective designs that assess engagement before and during implementation would be needed to establish whether these attitudes precede adoption. Fourth, some patterns, in particular Pattern 1, comprised few practices, which limits the precision and stability of the corresponding pattern-specific estimates and constrains generalisation (the implications of this imprecision for the cluster-specific intervention effects are discussed above). Relatedly, the cross-sectional design cannot capture temporal dynamics in adoption. Practices that engaged intensively early on may have experienced greater workflow disruption and could have disengaged over time, which would attenuate later effectiveness. Longitudinal, multi-site data would be required to examine such trajectories. Fifth, the study was conducted in a single German region (Westphalia-Lippe), which limits generalisability to other healthcare systems and regulatory contexts; replication elsewhere would help establish how far the identified patterns generalise. Sixth, the Silhouette index of 0.265 indicates moderate cluster separation. This implies that the adoption patterns are not sharply delineated and that the boundaries between them are partly fluid, which is likely to contribute to the limited classification performance of the prediction models. The patterns are therefore best understood as broad, overlapping types rather than discrete categories. Alternative distance metrics (Manhattan distance) and linkage methods (complete, average) yielded comparable solutions, supporting the robustness of the chosen classification. Seventh, the predictive performance of the Random Forest models without the survey indicator was modest (AUC 0.56–0.66), indicating that routinely available structural data alone do not reliably distinguish implementation behaviour. Because these analyses were exploratory and not based on pre-specified hypotheses, the observed associations, including the limited role of structural practice characteristics, should be interpreted as hypothesis-generating rather than confirmatory. Eighth, the study period extended into the COVID-19 pandemic, which may have affected implementation trajectories, for example through changes in consultation patterns and practice workload, in ways not captured by our analysis. Findings should therefore be read in this specific temporal context. Furthermore, cluster-specific intervention effects appeared more homogeneous in the pre-pandemic sensitivity analysis than in the main analysis. This may partly reflect reduced variability in hospitalisation and mortality outcomes before the onset of the COVID-19 pandemic, which likely affected healthcare utilisation and outcome trajectories during later study phases.

## Conclusions

This study shows that CDSS adoption in primary care is characterised by substantial heterogeneity that remains invisible when adopters and non-adopters are treated as homogeneous groups. We identified four distinct adoption patterns within a single trial context, differing in their implementation outcomes and in their cluster-specific intervention effects. That higher usage intensity did not correspond to larger cluster-specific intervention effects is a hypothesis-generating observation rather than a demonstrated effect, given the small number of practices in the most effective pattern and the uncertainty of the underlying estimates. It nonetheless suggests that implementation strategies should accommodate rather than suppress variation in adoption behaviour. Structural variables routinely available in administrative datasets were insufficient to distinguish adoption patterns, underscoring the value of assessing attitudinal and motivational factors as part of implementation monitoring. Together, these findings add to evidence that tailored, pattern-sensitive approaches to implementation may be preferable to uniform strategies.

## Supplementary Information

Below is the link to the electronic supplementary material.

Supplementary Material 1

## Data Availability

Claims data used for this study cannot be made available for public access to the BARMER science data warehouse due to data protection restrictions and confidentiality agreements. In accordance with the German Social Security Code (§ 75 SGB X), access to the data warehouse for research proposes can be requested from BARMER Statutory Health Insurance Fund, Wuppertal, Germany, if these data are required for research projects on welfare benefits or for scientific research on the labor market and occupation.

## Abbreviations

AdAM: Anwendung für ein digital gestütztes Arzneimitteltherapie-und Versorgungsmanagement
ATC: Anatomical Therapeutic Chemical
AUC: Area under the curve
BSNR: Betriebsstättennummer, operating site number
CDSS: Clinical decision support system
eHealth COMPATH: Partizipatives Mixed-Method Design zur Adaption und Evaluation eines Concept Mapping Ansatzes zur Entwicklung von Implementierungsstrategien für eHealth-Lösungen in der Patientenversorgung
eMMa: Electronic medication management; name of the software utilized in AdAM and developed by RpDoc^®^ Solutions GmbH
G-NoMAD: German Normalisation Process Theory
GP: General practitioner
HBSNR: Hauptbetriebsstättennummer, main operating site number
IQR: Interquartil range
KVWL: Kassenärztliche Vereinigung Westfalen Lippe, the regional Association of Statutory Health Insurance Physicians
LANR: Lebenslange Arztnummer, the lifetime physician number
ORIC: Organizational readiness for implementing change
PAR: Practice Adaptive Reserve
ROC: Receiver operating characteristic
SD: Standard deviation
SW-CRT: Cluster-randomised stepped wedge trial
W-DWH: Wissenschaftliches Data Ware House
