# Supplementary material for: Patterns of CDSS adoption in primary care: a cluster analysis and predictive modelling study from a stepped wedge trial
Source: Implement Sci. 2026 Jun 18;21:38. doi: 10.1186/s13012-026-01516-0 (PMC13281615; doi:10.1186/s13012-026-01516-0)
Supplement: Supplementary file 1 — Supplementary Material 1 [file 13012_2026_1516_MOESM1_ESM.docx]

## Supplementary Material

Figure S1 Schematic working process for GPs that use eMMa [21].


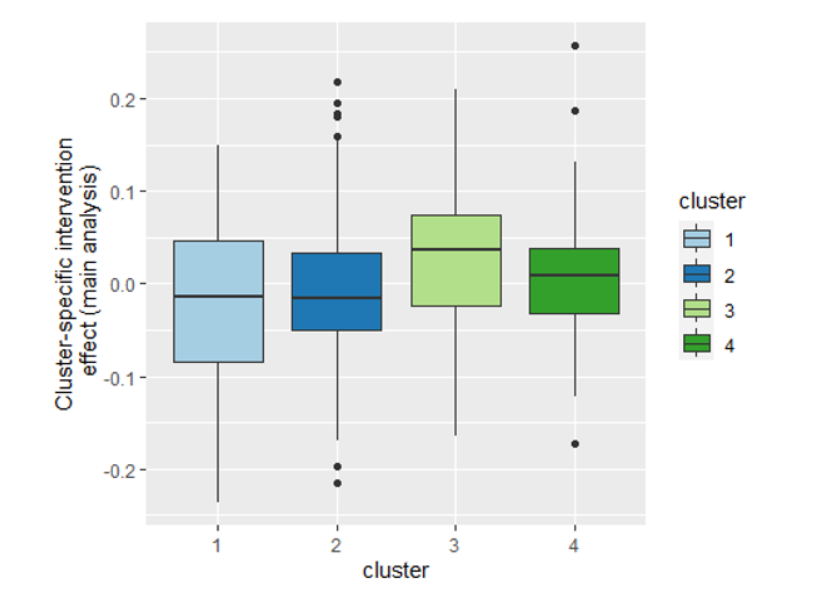


Figure S2 Boxplot of the cluster-specific intervention effect for the combined endpoint from the main analysis of the AdAM study across all clusters.


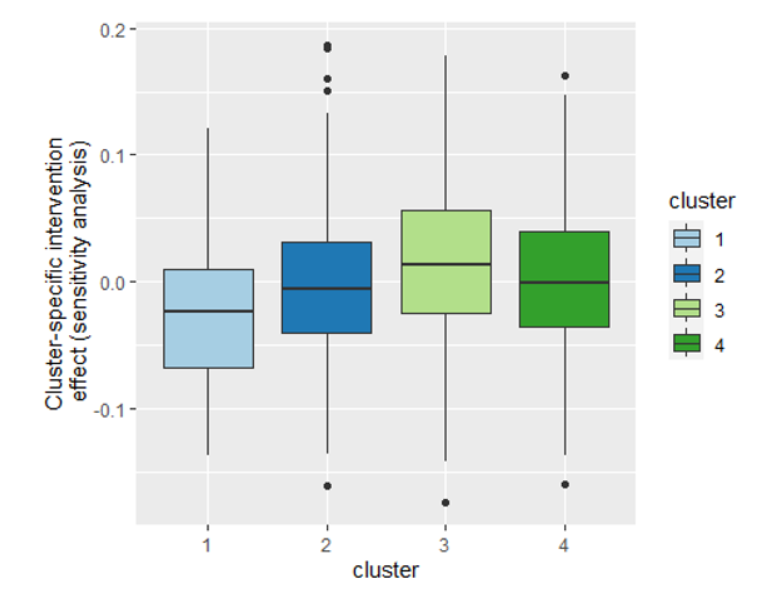


Figure S3 Boxplot of the cluster-specific intervention effect for the combined endpoint from a sensitivity analysis of the AdAM study across all clusters.


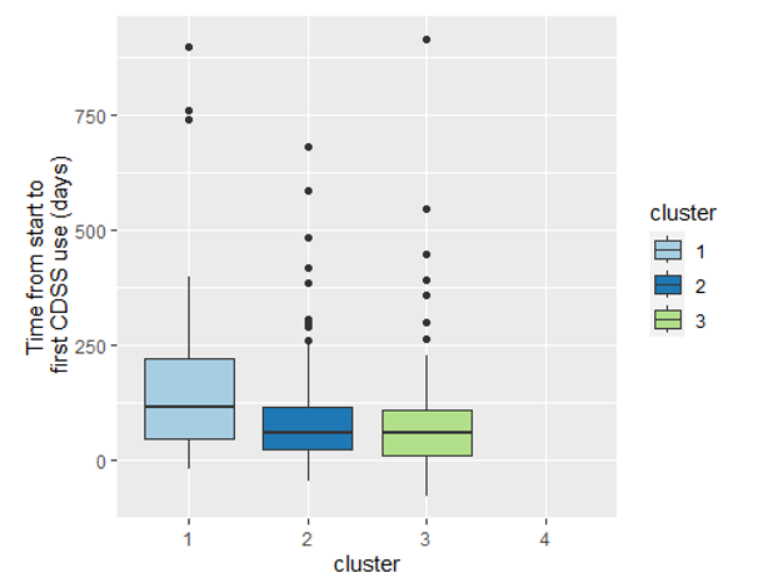


Figure S4 Boxplot of the time from the start of primary care practice participation to the first CDSS use in days (early versus late adoption) across all clusters.


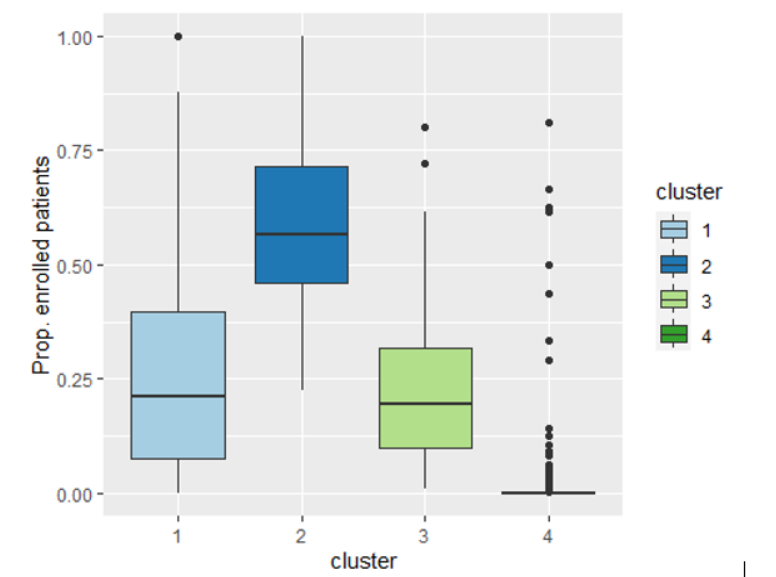


Figure S5 Boxplots of the proportion of enrolled patients to potentially eligible patients (penetration) across all clusters.


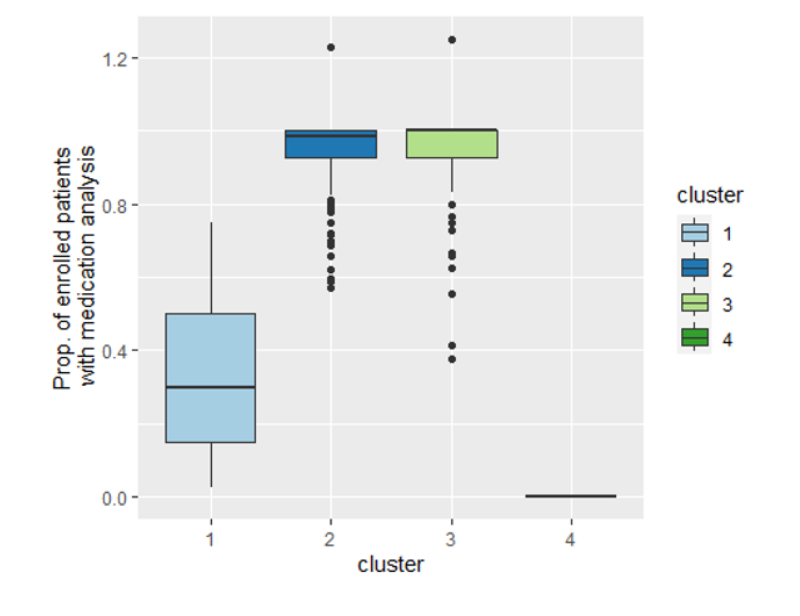


Figure S6 Boxplot of the proportion of enrolled patients with medication review (fidelity) across all clusters.


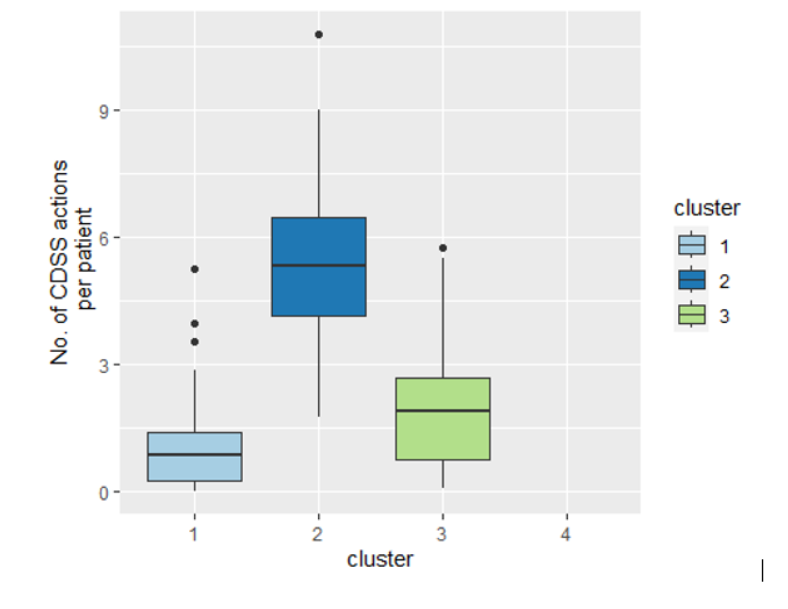


Figure S7 Boxplot of the average number of CDSS actions performed per patient prescribed in the AdAM study and treated in the corresponding primary care practice (intensity) across all clusters.

Table S1: List and description of candidate variables for adoption pattern classification and their availability.

| **Candidate explanatory variables** | **Description** | **Available in Pattern 1-3 (in %)** | **Available in all patterns (in %)** |
| --- | --- | --- | --- |
| Data source: Routine data from a major German health insurance company (BARMER) | | | |
| Mean age of patients | Mean age of all patients of the primary care practice during intervention phase | 100 | 100 |
| Proportion of female patients | Proportion of female patients of the primary care practice during intervention phase | 100 | 100 |
| Mean level of nursing care of patients | Mean level of nursing care of all patients of the primary care practice during intervention phase | 100 | 100 |
| Mean medication-related chronic disease prognostic score (medCDS) of patients | Mean medication-related chronic disease prognostic score (medCDS) of all patients of the primary care practice during intervention phase | 100 | 100 |
| Data source: structural data from the primary care practices involved in AdAM (KVWL) | | | |
| Practice type | Single-handed practice, group practice, or ambulatory healthcare centers | 100 | 100 |
| Panel size | Divided into quintiles and only considering consultations with GPs:  1-870  871-1163  1164-1468  1469-2027  ≥ 2028 | 100 | 100 |
| Age of GP | If there are more than one GP in practice, we consider the mean. | 100 | 100 |
| Proportion of female GPs | If there are more than one GP in practice, we consider the proportion of female GPs. | 100 | 100 |
| Specialization of physician | Specialised in general practice, internist active in general practice, no specialist qualification or other | 100 | 100 |
| Member of GP network (*Arztnetz*) |  | 100 | 100 |
| Data source: data from a cross-sectional postal survey of GPs participating in AdAM (Center for Health Economics and Health Services Research at the University of Wuppertal) | | | |
| Indicator whether the survey has been completed by at least one GP in the practice |  | 100 | 100 |
| A: On the use of AdAM software in general | For more details, see Table S2. | Approx. 60 | Approx. 35 |
| B1: On the relevance of the topic of polypharmacy | For more details, see Table S2. | Approx. 50 | Approx. 29 |
| B2: Dealing with change in your primary care practice / Practice Adaptive Reserve (PAR) measure | For more details, see Table S2. The PAR measure assesses a practice’s capacity to adapt to changes. | Approx. 49 | Approx. 28 |
| C: Introducing (implementing) AdAM software into your daily practice / Organisational readiness for implementing change (ORIC) measure | For more details, see Table S2. The ORIC measures an organization’s preparedness to adopt changes. It comprises two subscales:   1. **Change Commitment**: This subscale evaluates the resolve, motivation, and willingness of involved parties to implement the change. 2. **Change Efficacy**: This subscale assesses the confidence of involved parties in their ability to execute necessary tasks, overcome challenges, and coordinate required efforts. | Approx. 50 | Approx. 28 |
| D: Using AdAM software in your daily practice / German Normalisation Process Theory (G-NoMAD) measure | For more details, see Table S2**.** The G-NoMAD is a tool designed to assess the degree to which an intervention becomes routinely embedded in everyday practice, a process known as normalization. It consists of four subscales that capture different dimensions of normalization:   1. **Coherence**: This subscale measures the extent to which the intervention makes sense to the individuals involved, and how well it aligns with their existing values, beliefs, and practices. 2. **Cognitive Participation**: This subscale assesses the level of engagement and commitment among the individuals involved in the intervention, including their willingness to participate and the extent to which they collectively support the intervention. 3. **Collective Action**: This subscale evaluates the practical implementation of the intervention, including the work required to integrate it into everyday practice, the allocation of resources, and the management of any challenges or obstacles that arise. 4. **Reflexive Monitoring**: This subscale measures the extent to which the intervention is subject to ongoing evaluation and reflection, including the assessment of its impacts, the identification of any problems or issues, and the consideration of alternative approaches or modifications. | Approx. 49 | Approx. 27 |
| E: Summary | For more details, see Table S2. | Approx. 50 | Approx. 27 |

Table S2: Descriptive characteristics of primary care practices, general practitioners, and questionnaire-based measures across adoption patterns. Continuous variables are presented as median [IQR]. P-values for continuous variables were calculated using Kruskal–Wallis tests; p-values for categorical variables were calculated using chi-squared tests.

| **Candidate explanatory variables** | **Pattern 1**  **(n=43)** | **Pattern 2 (n=213)** | **Pattern 3 (n=100)** | **Pattern 4 (n=368)** | **p-value (Pattern 1-4)** | **p-value (Pattern 1-3)** |
| --- | --- | --- | --- | --- | --- | --- |
| **Mean age of patients** | 73.2  [70.7, 75.7] | 73.14 [70.9, 75.7] | 73.1  [70.8, 75.3] | 72.9  [70.0, 75.5] | 0.484 | 0.822 |
| **Proportion of female patients** | 0.66  [0.55, 0.73] | 0.62  [0.55, 0.70] | 0.61  [0.56, 0.66] | 0.62  [0.55, 0.69 | 0.282 | 0.135 |
| **Mean level of nursing care of patients** | 1.78  [1.61, 1.97] | 1.78  [1.59, 1.95] | 1.73  [1.57, 1.91] | 1.77  [1.55, 2.01] | 0.729 | 0.560 |
| **Mean medication-related chronic disease prognostic score (medCDS) of patients** | 0.13  [0.06, 0.16] | 0.12  [0.09,  0.16] | 0.11  [0.08, 0.16] | 0.11  [0.07, 1.17] | 0.743 | 0.712 |
| **Practice type** |  |  |  |  | **0.004** | **0.025** |
| Single-handed practice | 23  (52.5%) | 133 (62.4%) | 45  (45.0%) | 244 (66.3%) |  |  |
| Group practice | 20  (46.5%) | 79  (37.1%) | 53  (53.0%) | 121 (32.9%) |  |  |
| Ambulatory healthcare centres | 0  (0.0%) | 1  (0.47%) | 2  (2.0%) | 3  (0.82%) |  |  |
| **Panel size** |  |  |  |  | **<0.001** | **<0.001** |
| 1 – 870 | 1  (2.3%) | 27  (12.7%) | 6  (6.0%) | 45  (12.2%) |  |  |
| 871 – 1163 | 11  (25.6%) | 39  (18.3%) | 14  (14.0%) | 58  (15.8%) |  |  |
| 1164 – 1468 | 5  (11.6%) | 49  (23.0%) | 11  (11.0%) | 75  (20.4%) |  |  |
| 1469 – 2027 | 13  (30.2%) | 51  (23.9%) | 29  (29.0%) | 89  (24.2%) |  |  |
| ≥ 2028 | 13  (30.2%) | 47  (22.1%) | 40  (40.0%) | 100 (27.2%) |  |  |
| **Age of GP** | 54.7  [49.5, 60.0] | 54.0  [49.0, 59.0] | 53.8  [50.8, 58.0] | 56.0  [50.9, 62.0] | **0.002** | 0.798 |
| **Proportion of female GPs** | 0  [0, 0.5] | 0.33  [0,1] | 0  [0, 0.5] | 0  [0, 0.75] | **0.001** | **0.002** |
| **Specialization of physician** |  |  |  |  | **<0.001** | **<0.001** |
| Internist active in primary care practice | 20  (46.5%) | 57  (26.8%) | 29  (29.0%) | 107 (29.1%) |  |  |
| Specialised in general practice | 14  (32.6%) | 120 (56.3%) | 51  (51.0%) | 224 (60.9%) |  |  |
| No specialist qualification | 0  (0.0%) | 4  (1.88%) | 2  (2.0%) | 16  (4.35%) |  |  |
| Different specializations | 9  (20.9%) | 32  (15.0%) | 18  (18.0%) | 19  (5.16%) |  |  |
| **Member of GP network (*Arztnetz*)** | 14  (32.6%) | 68  (31.9%) | 24  (24.0%) | 105 (28.5%) | 0.496 | 0.329 |
| **Survey has been completed by at least one GP in the practice** | 17  (39.5%) | 158 (74.2%) | 60  (60.0%) | 38  (10.3%) | **<0.001** | **<0.001** |
| **Data source: data from a cross-sectional postal survey of GPs participating in AdAM (Center for Health Economics and Health Services Research at the University of Wuppertal)** |  |  |  |  |  |  |
| **A: On the use of AdAM software in general** |  |  |  |  |  |  |
| **Did you participate in AdAM training during the project period? (A3)** |  |  |  |  | **0.045** | 0.602 |
| Yes | 7  (16.3%) | 82  (38.5%) | 33  (33.0%) | 11  (3.0%) |  |  |
| No | 10  (23.3%) | 75  (35.2%) | 27  (27.0%) | 27  (7.3%) |  |  |
| No information provided | 26  (60.5%) | 56  (26.3%) | 40  (40.0%) | 330  (89.7%) |  |  |
| **Has any of your staff participated in an AdAM training? (A4)** |  |  |  |  | 0.088 | 0.943 |
| Yes | 4  (9.3%) | 46  (21.6%) | 18  (18.0%) | 4  (1.1%) |  |  |
| No | 13  (30.2%) | 111  (52.1%) | 42  (42.0%) | 34  (9.2%) |  |  |
| No information provided | 26  (60.5%) | 56  (26.3%) | 40  (40.0%) | 330  (89.7%) |  |  |
| **Are the employees at your practice familiar with the AdAM software? (A6)** |  |  |  |  | **0.024** | 0.942 |
| Yes | 16  (37.2%) | 140  (65.7%) | 54  (54.0%) | 25  (6.8%) |  |  |
| No | 1  (2.3%) | 18  (8.5%) | 6  (6.0%) | 11  (3.0%) |  |  |
| No information provided | 26  (60.5%) | 55  (25.8%) | 40  (40.0%) | 332  (90.2%) |  |  |
| **Does your practice management system have a decision support function for use with multiple medications, such as providing warnings about drug interactions? (A7)** |  |  |  |  | **<0.001** | 0.713 |
| **Yes, and I use it** | 11  (25.6%) | 69  (32.4%) | 23  (23.0%) | 18  (4.9%) |  |  |
| **Yes, but I don’t use it** | 1  (2.3%) | 20  (9.4%) | 11  (11.0%) | 1  (0.3%) |  |  |
| **I don’t know** | 1  (2.3%) | 15  (7.0%) | 6  (6.0%) | 2  (0.5%) |  |  |
| **No, it doesn’t** | 4  (9.3%) | 48  (22.5%) | 19  (19.0%) | 14  (3.8%) |  |  |
| **No information provided** | 26  (60.5%) | 61  (28.6%) | 41  (41.0%) | 333  (90.5%) |  |  |
| **Do you use any additional decision support tools (apps, checklists, guidelines, etc.)? (A8)** |  |  |  |  | 0.528 | 0.683 |
| Yes | 16  (37.2%) | 142 (66.7%) | 52  (52.0%) | 28  (7.6%) |  |  |
| No | 1  (2.3%) | 16  (7.5%) | 8  (8.0%) | 6  (1.6%) |  |  |
| No information provided | 26  (60.5%) | 55  (25.8%) | 40  (40.0%) | 334 (90.8%) |  |  |
| **B1: On the relevance of the topic of polypharmacy** |  |  |  |  |  |  |
| **Polypharmacy is a very important topic in my daily practice. (B1.1)** |  |  |  |  | **<0.001** | 0.504 |
| Strongly disagree | 0  (0.0%) | 0  (0.0%) | 0  (0.0%) | 0  (0.0%) |  |  |
| Disagree | 0  (0.0%) | 2  (0.9%) | 2  (2.0%) | 0  (0.0%) |  |  |
| Neither agree nor disagree | 0  (0.0%) | 14  (6.6%) | 5  (5.0%) | 3  (0.8%) |  |  |
| Agree | 5  (11.6%) | 37  (17.4%) | 13  (13.0%) | 9  (2.4%) |  |  |
| Strongly agree | 7  (16.3%) | 77  (36.2%) | 18  (18.0%) | 19  (5.2%) |  |  |
| No information provided | 31  (72.1%) | 83  (39.0%) | 62  (62.0%) | 337  (91.6%) |  |  |
| **In my opinion, polypharmacy is also a high-priority issue in other practices. (B1.2)** |  |  |  |  | **<0.001** | 0.597 |
| Strongly disagree | 0  (0.0%) | 1  (0.5%) | 0  (0.0%) | 0  (0.0%) |  |  |
| Disagree | 0  (0.0%) | 4  (1.9%) | 3  (3.0%) | 0  (0.0%) |  |  |
| Neither agree nor disagree | 2  (4.7%) | 31  (14.6%) | 13  (13.0%) | 5  (1.4%) |  |  |
| Agree | 5  (11.6%) | 45  (21.1%) | 13  (13.0%) | 10  (2.7%) |  |  |
| Strongly agree | 5  (11.6%) | 47  (22.1%) | 9  (9.0%) | 15  (4.1%) |  |  |
| No information provided | 31  (72.1%) | 85  (39.9%) | 62  (62.0%) | 338  (91.8%) |  |  |
| **My patients are keen to ask questions about side effects and interactions. (B1.3)** |  |  |  |  | **<0.001** | 0.589 |
| Strongly disagree | 0  (0.0%) | 0  (0.0%) | 1  (1.0%) | 1  (0.3%) |  |  |
| Disagree | 0  (0.0%) | 14  (6.6%) | 5  (5.0%) | 5  (1.4%) |  |  |
| Neither agree nor disagree | 5  (11.6%) | 36  (16.9%) | 11  (11.0%) | 5  (1.4%) |  |  |
| Agree | 6  (14.0%) | 58  (27.3%) | 16  (16.0%) | 10  (2.7%) |  |  |
| Strongly agree | 1  (2.3%) | 22  (10.3%) | 4  (4.0%) | 10  (2.7%) |  |  |
| No information provided | 31  (72.1%) | 83  (39.0%) | 63  (63.0%) | 337  (91.6%) |  |  |
| **In recent years, the issue of polypharmacy has become increasingly important in my daily treatment activities. (B1.4)** |  |  |  |  | **<0.001** | 0.554 |
| Strongly disagree | 0  (0.0%) | 1  (0.5%) | 0  (0.0%) | 0  (0.0%) |  |  |
| Disagree | 0  (0.0%) | 3  (1.4%) | 3  (3.0%) | 1  (0.3%) |  |  |
| Neither agree nor disagree | 1  (2.3%) | 12  (5.6%) | 1  (1.0%) | 7  (1.9%) |  |  |
| Agree | 4  (9.3%) | 58  (27.2%) | 18  (18.0%) | 7  (1.9%) |  |  |
| Strongly agree | 7  (16.3%) | 56  (26.3%) | 15  (15.0%) | 16  (4.3%) |  |  |
| No information provided | 31  (72.1%) | 83  (39.0%) | 63  (63.0%) | 337  (91.6%) |  |  |
| **Our employees are extremely knowledgeable about the risks associated with polypharmacy. (B1.5)** |  |  |  |  | **<0.001** | 0.426 |
| Strongly disagree | 0  (0.0%) | 1  (0.5%) | 2  (2.0%) | 1  (0.3%) |  |  |
| Disagree | 1  (2.3%) | 23  (10.8%) | 4  (4.0%) | 2  (0.5%) |  |  |
| Neither agree nor disagree | 7  (16.3%) | 49  (23.0%) | 15  (15.0%) | 11  (3.0%) |  |  |
| Agree | 3  (7.0%) | 51  (23.9%) | 14  (14.0%) | 13  (3.5%) |  |  |
| Strongly agree | 1  (2.3%) | 6  (2.8%) | 3  (3.0%) | 4  (1.1%) |  |  |
| No information provided | 32  (74.4%) | 83  (39.0%) | 62  (62.0%) | 337  (91.6%) |  |  |
| **The practice team can use technology to assess the risks of polypharmacy. (B1.6)** |  |  |  |  | **<0.001** | 0.566 |
| Strongly disagree | 0  (0.0%) | 1  (0.5%) | 0  (0.0%) | 0  (0.0%) |  |  |
| Disagree | 0  (0.0%) | 8  (3.8%) | 2  (2.0%) | 4  (1.1%) |  |  |
| Neither agree nor disagree | 4  (9.3%) | 25  (11.7%) | 5  (5.0%) | 4  (1.1%) |  |  |
| Agree | 7  (16.2%) | 63  (29.6%) | 17  (17.0%) | 9  (2.4%) |  |  |
| Strongly agree | 1  (2.3%) | 33  (15.5%) | 14  (14.0%) | 12  3.3%) |  |  |
| No information provided | 31  (72.1%) | 83  (39.0%) | 62  (62.0%) | 339  (92.1%) |  |  |
| **B2: Dealing with change in your primary care practice / Practice Adaptive Reserve (PAR) measure** |  |  |  |  |  |  |
| **We regularly take the time to consider how we can improve our processes. (B2.1)** |  |  |  |  | **<0.001** | 0.456 |
| Strongly disagree | 0  (0.0%) | 1  (0.5%) | 0  (0.0%) | 0  (0.0%) |  |  |
| Disagree | 0  (0.0%) | 3  (1.4%) | 3  (3.0%) | 1  (0.3%) |  |  |
| Neither agree nor disagree | 1  (2.3%) | 16  (7.5%) | 5  (5.0%) | 1  (0.3%) |  |  |
| Agree | 5  (11.6%) | 63  (29.6%) | 12  (12.0%) | 14  (3.8%) |  |  |
| Strongly agree | 6  (14.0%) | 46  (3.0%) | 18  (18.0%) | 15  (4.1%) |  |  |
| No information provided | 31  (72.1%) | 84  (39.4%) | 62  (62.0%) | 337  (91.6%) |  |  |
| **The staff at our practice are always looking for new ways to improve their approach. (B2.2)** |  |  |  |  | **<0.001** | 0.672 |
| Strongly disagree | 0  (0.0%) | 1  (0.5%) | 0  (0.0%) | 0  (0.0%) |  |  |
| Disagree | 1  (2.3%) | 4  (1.9%) | 2  (2.0%) | 2  (0.5%) |  |  |
| Neither agree nor disagree | 2  (4.7%) | 31  (14.6%) | 9  (9.0%) | 7  (1.9%) |  |  |
| Agree | 8  (18.6%) | 58  (27.2%) | 17  (17.0%) | 12  (3.3%) |  |  |
| Strongly agree | 1  (2.3%) | 36  (16.9%) | 10  (10.0%) | 10  (2.7%) |  |  |
| No information provided | 31  (72.1%) | 83  (39.0%) | 62  (62.0%) | 337  (91.6%) |  |  |
| **Employees at this practice are encouraged to speak openly about what is and isn’t working well. (B2.3)** |  |  |  |  | **<0.001** | 0.916 |
| Strongly disagree | 0  (0.0%) | 1  (0.5%) | 0  (0.0%) | 0  (0.0%) |  |  |
| Disagree | 0  (0.0%) | 3  (1.4%) | 0  (0.0%) | 0  (0.0%) |  |  |
| Neither agree nor disagree | 2  (4.7%) | 10  (4.7%) | 4  (4.0%) | 3  (0.8%) |  |  |
| Agree | 6  (14.0%) | 70  (32.9%) | 21  (21.0%) | 13  (3.5%) |  |  |
| Strongly agree | 4  (9.3%) | 46  (21.6%) | 13  (13.0%) | 15  (4.1%) |  |  |
| No information provided | 31  (72.1%) | 83  (39.0%) | 62  (62.0%) | 337  (91.6%) |  |  |
| **The employees at this practice are aware of how their actions affect others. (B2.4)** |  |  |  |  | **<0.001** | 0.726 |
| Strongly disagree | 0  (0.0%) | 1  (0.5%) | 0  (0.0%) | 0  (0.0%) |  |  |
| Disagree | 0  (0.0%) | 3  (1.4%) | 1  (1.0%) | 0  (0.0%) |  |  |
| Neither agree nor disagree | 2  (4.7%) | 24  (11.3%) | 7  (7.0%) | 7  (1.9%) |  |  |
| Agree | 9  (20.9%) | 60  (28.2%) | 19  (19.0%) | 11  (3.0%) |  |  |
| Strongly agree | 1  (2.3%) | 41  (19.2%) | 11  (11.0%) | 13  (3.5%) |  |  |
| No information provided | 31  (72.1%) | 84  (39.4%) | 62  (62.0%) | 337  (91.6%) |  |  |
| **Most employees at this practice are willing to consider feedback from others and adapt their behaviour accordingly. (B2.5)** |  |  |  |  | **<0.001** | 0.344 |
| Strongly disagree | 0  (0.0%) | 1  (0.5%) | 0  (0.0%) | 0  (0.0%) |  |  |
| Disagree | 0  (0.0%) | 4  (1.9%) | 0  (0.0%) | 0  (0.0%) |  |  |
| Neither agree nor disagree | 3  (7.0%) | 14  (6.6%) | 5  (5.0%) | 5  (1.4%) |  |  |
| Agree | 9  (20.9%) | 75  (35.2%) | 23  (23.0%) | 17  (4.6%) |  |  |
| Strongly agree | 0  (0.0%) | 36  (16.9%) | 10  (10.0%) | 9  (2.4%) |  |  |
| No information provided | 31  (72.1%) | 83  (39.0%) | 62  (62.0%) | 337  (91.6%) |  |  |
| **The practice actively encourages all its employees to share their ideas. (B2.6)** |  |  |  |  | 0.494 | 0.698 |
| Strongly disagree | 0  (0.0%) | 1  (0.5%) | 0  (0.0%) | 0  (0.0%) |  |  |
| Disagree | 0  (0.0%) | 1  (0.5%) | 0  (0.0%) | 0  (0.0%) |  |  |
| Neither agree nor disagree | 0  (0.0%) | 13  (6.1%) | 3  (3.0%) | 0  (0.0%) |  |  |
| Agree | 5  (11.6%) | 56  (26.3%) | 22  (22.0%) | 13  (3.5%) |  |  |
| Strongly agree | 7  (16.3%) | 59  (27.7%) | 13  (13.0%) | 18  (4.9%) |  |  |
| No information provided | 31  (72.1%) | 83  (39.0%) | 62  (62.0%) | 337  (91.6%) |  |  |
| **I trust that all the other employees at this practice are doing their jobs well. (B2.7)** |  |  |  |  | **<0.001** | 0.777 |
| Strongly disagree | 0  (0.0%) | 1  (0.5%) | 0  (0.0%) | 0  (0.0%) |  |  |
| Disagree | 0  (0.0%) | 2  (0.9%) | 0  (0.0%) | 1  (0.4%) |  |  |
| Neither agree nor disagree | 2  (4.7%) | 12  (5.6%) | 2  (2.0%) | 5  (1.4%) |  |  |
| Agree | 7  (16.3%) | 64  (30.0%) | 18  (18.0%) | 13  (3.5%) |  |  |
| Strongly agree | 3  (7.0%) | 51  (23.9%) | 18  (18.0%) | 12  (3.3%) |  |  |
| No information provided | 31  (72.1%) | 83  (39.0%) | 62  (62.0%) | 337  (91.6%) |  |  |
| **This practice solves difficult problems through personal conversations. (B2.8)** |  |  |  |  | 0.853 | 0.844 |
| Strongly disagree | 0  (0.0%) | 1  (0.5%) | 0  (0.0%) | 0  (0.0%) |  |  |
| Disagree | 0  (0.0%) | 1  (0.5%) | 0  (0.0%) | 0  (0.0%) |  |  |
| Neither agree nor disagree | 0  (0.0%) | 7  (3.3%) | 2  (2.0%) | 0  (0.0%) |  |  |
| Agree | 4  (9.3%) | 58  (27.2%) | 20  (20.0%) | 12  (3.3%) |  |  |
| Strongly agree | 8  (18.6%) | 65  (30.5%) | 16  (16.0%) | 18  (4.9%) |  |  |
| No information provided | 31  (72.1%) | 81  (38.0%) | 62  (62.0%) | 338  (91.8%) |  |  |
| **We regularly take the time to think about the way we do things. (B2.9)** |  |  |  |  | **<0.001** | 0.805 |
| Strongly disagree | 0  (0.0%) | 1  (0.5%) | 0  (0.0%) | 0  (0.0%) |  |  |
| Disagree | 0  (0.0%) | 4  (1.9%) | 1  (1.0%) | 1  (0.4%) |  |  |
| Neither agree nor disagree | 0  (0.0%) | 20  (9.4%) | 6  (6.0%) | 5  (1.4%) |  |  |
| Agree | 5  (11.6%) | 61  (28.6%) | 17  (17.0%) | 8  (2.3%) |  |  |
| Strongly agree | 7  (16.3%) | 45  (21.1%) | 14  (14.0%) | 16  (4.3%) |  |  |
| No information provided | 31  (72.1%) | 82  (38.5%) | 62  (62.0%) | 338  (91.8%) |  |  |
| **When we try something new, we spend time reflecting on how it went. (B2.10)** |  |  |  |  | **<0.001** | 0.934 |
| Strongly disagree | 0  (0.0%) | 2  (0.9%) | 0  (0.0%) | 0  (0.0%) |  |  |
| Disagree | 0  (0.0%) | 4  (1.9%) | 1  (1.0%) | 0  (0.0%) |  |  |
| Neither agree nor disagree | 1  (2.3%) | 15  (7.0%) | 6  (6.0%) | 8  (2.2%) |  |  |
| Agree | 5  (11.6%) | 69  (32.4%) | 18  (18.0%) | 10  (2.7%) |  |  |
| Strongly agree | 6  (14.0%) | 41  (19.2%) | 13  (13.0%) | 12  (3.3%) |  |  |
| No information provided | 31  (72.1%) | 82  (38.5%) | 62  (62.0%) | 338  (91.8%) |  |  |
| **I will make sure that we have enough time and space to discuss how we can improve care. (B2.11)** |  |  |  |  | **<0.001** | 0.459 |
| Strongly disagree | 0  (0.0%) | 1  (0.5%) | 0  (0.0%) | 0  (0.0%) |  |  |
| Disagree | 0  (0.0%) | 4  (1.9%) | 3  (3.0%) | 0  (0.0%) |  |  |
| Neither agree nor disagree | 0  (0.0%) | 21  (9.9%) | 9  (9.0%) | 8  (2.2%) |  |  |
| Agree | 8  (18.6%) | 73  (34.3%) | 18  (18.0%) | 10  (2.7%) |  |  |
| Strongly agree | 4  (9.3%) | 32  (15.0%) | 8  (8.0%) | 11  (3.0%) |  |  |
| No information provided | 31  (72.1%) | 82  (38.5%) | 62  (62.0%) | 339  (92.1%) |  |  |
| **I create an environment in which people can achieve things. (B2.12)** |  |  |  |  | **<0.001** | 0.832 |
| Strongly disagree | 0  (0.0%) | 1  (0.5%) | 0  (0.0%) | 0  (0.0%) |  |  |
| Disagree | 0  (0.0%) | 2  (0.9%) | 0  (0.0%) | 0  (0.0%) |  |  |
| Neither agree nor disagree | 0  (0.0%) | 13  (6.1%) | 5  (5.0%) | 3  (0.8%) |  |  |
| Agree | 9  (20.9%) | 68  (31.9%) | 21  (21.0%) | 14  (3.8%) |  |  |
| Strongly agree | 3  (7.0%) | 47  (22.1%) | 12  (12%) | 13  (3.5%) |  |  |
| No information provided | 31  (72.1%) | 82  (38.5%) | 62  (62.0%) | 338  (91.8%) |  |  |
| **I promote an enjoyable working environment. (B2.13)** |  |  |  |  | 0.845 | 0.901 |
| Strongly disagree | 0  (0.0%) | 1  (0.5%) | 0  (0.0%) | 0  (0.0%) |  |  |
| Disagree | 0  (0.0%) | 1  (0.5%) | 0  (0.0%) | 0  (0.0%) |  |  |
| Neither agree nor disagree | 1  (0.2%) | 5  (2.3%) | 1  (1.0%) | 1  (0.3%) |  |  |
| Agree | 6  (14.0%) | 65  (30.5%) | 21  (21.0%) | 11  (3.0%) |  |  |
| Strongly agree | 5  (11.6%) | 58  (27.2%) | 16  (16.0%) | 18  (4.9%) |  |  |
| No information provided | 31  (72.1%) | 83  (39.0%) | 62  (62.0%) | 338  (91.8%) |  |  |
| **I strongly support the efforts to change this practice. (B2.14)** |  |  |  |  | **<0.001** | 0.641 |
| Strongly disagree | 0  (0.0%) | 1  (0.5%) | 0  (0.0%) | 0  (0.0%) |  |  |
| Disagree | 0  (0.0%) | 1  (0.5%) | 2  (2.0%) | 1  (0.3%) |  |  |
| Neither agree nor disagree | 1  (0.2%) | 9  (4.2%) | 1  (1.0%) | 4  (1.1%) |  |  |
| Agree | 6  (14.0%) | 57  (26.8%) | 17  (17.0%) | 9  (2.4%) |  |  |
| Strongly agree | 5  (11.6%) | 63  (20.6%) | 18  (18.0%) | 16  (4.3%) |  |  |
| No information provided | 31  (72.1%) | 82  (38.5%) | 62  (62.0%) | 338  (91.8%) |  |  |
| **In this practice, they learn from their mistakes. (B2.15)** |  |  |  |  | **<0.001** | 0.971 |
| Strongly disagree | 0  (0.0%) | 1  (0.5%) | 0  (0.0%) | 0  (0.0%) |  |  |
| Disagree | 0  (0.0%) | 1  (0.5%) | 0  (0.0%) | 0  (0.0%) |  |  |
| Neither agree nor disagree | 1  (0.2%) | 17  (8.0%) | 4  (4.0%) | 6  (1.6%) |  |  |
| Agree | 7  (16.3%) | 58  (27.2%) | 18  (18.0%) | 9  (2.4%) |  |  |
| Strongly agree | 4  (9.3%) | 55  (25.8%) | 16  (16.0%) | 15  (4.1%) |  |  |
| No information provided | 31  (72.1%) | 81  (38.0%) | 62  (62.0%) | 338  (91.8%) |  |  |
| **It is difficult to make any changes to our practice. (B2.16)** |  |  |  |  | **<0.001** | 0.823 |
| Strongly disagree | 2  (4.7%) | 31  (14.6%) | 10  (10.0%) | 11  (3.0%) |  |  |
| Disagree | 9  (20.9%) | 59  (27.7%) | 18  (18.0%) | 11  (3.0%) |  |  |
| Neither agree nor disagree | 1  (2.3%) | 32  (15.0%) | 9  (9.0%) | 4  (1.1%) |  |  |
| Agree | 0  (0.0%) | 6  (2.8%) | 1  (1.0%) | 3  (0.8%) |  |  |
| Strongly agree | 0  (0.0%) | 4  (1.9%) | 0  (0.0%) | 1  (0.3%) |  |  |
| No information provided | 31  (72.1%) | 81  (38.0%) | 62  (62.0%) | 338  (91.8%) |  |  |
| **Positive changes have already been made as a result of mistakes. (B2.17)** |  |  |  |  | **<0.001** | 0.935 |
| Strongly disagree | 0  (0.0%) | 1  (0.5%) | 0  (0.0%) | 1  (0.3%) |  |  |
| Disagree | 0  (0.0%) | 2  (0.9%) | 0  (0.0%) | 1  (0.3%) |  |  |
| Neither agree nor disagree | 3  (7.0%) | 18  (8.5%) | 6  (6.0%) | 3  (0.8%) |  |  |
| Agree | 7  (16.3%) | 78  (36.6%) | 22  (22.0%) | 12  (3.3%) |  |  |
| Strongly agree | 2  (4.7%) | 33  (15.5%) | 10  (10.0%) | 13  (3.5%) |  |  |
| No information provided | 31  (72.1%) | 81  (38.0%) | 62  (62.0%) | 338  (91.8%) |  |  |
| **The employees at this practice have access to all the information they need to carry out their roles effectively. (B2.18)** |  |  |  |  | 0.482 | 0.434 |
| Strongly disagree | 0  (0.0%) | 0  (0.0%) | 0  (0.0%) | 0  (0.0%) |  |  |
| Disagree | 0  (0.0%) | 2  (0.9%) | 0  (0.0%) | 0  (0.0%) |  |  |
| Neither agree nor disagree | 0  (0.0%) | 11  (5.2%) | 0  (0.0%) | 2  (0.5%) |  |  |
| Agree | 10  (23.3%) | 82  (38.5%) | 27  (27.0%) | 16  (4.3%) |  |  |
| Strongly agree | 2  (4.7%) | 37  (17.4%) | 11  (11.0%) | 12  (3.3%) |  |  |
| No information provided | 31  (72.1%) | 81  (38.0%) | 62  (62.0%) | 338  (91.8%) |  |  |
| **If we encounter a problem in the practice, we make every effort to find out what the real issue is. (B2.19)** |  |  |  |  | 0.675 | 0.793 |
| Strongly disagree | 0  (0.0%) | 2  (0.9%) | 0  (0.0%) | 0  (0.0%) |  |  |
| Disagree | 0  (0.0%) | 1  (0.5%) | 0  (0.0%) | 0  (0.0%) |  |  |
| Neither agree nor disagree | 0  (0.0%) | 11  (5.2%) | 4  (4.0%) | 0  (0.0%) |  |  |
| Agree | 4  (9.3%) | 55  (25.8%) | 19  (19.0%) | 12  (3.3%) |  |  |
| Strongly agree | 8  (18.6%) | 63  (29.6%) | 15  (15.0%) | 18  (4.9%) |  |  |
| No information provided | 31  (72.1%) | 81  (38.0%) | 62  (62.0%) | 338  (91.8%) |  |  |
| **There are many opportunities for me to develop further in my work. (B2.20)** |  |  |  |  | **<0.001** | **0.030** |
| Strongly disagree | 0  (0.0%) | 1  (0.5%) | 0  (0.0%) | 0  (0.0%) |  |  |
| Disagree | 3  (7.0%) | 1  (0.5%) | 1  (1.0%) | 2  (0.5%) |  |  |
| Neither agree nor disagree | 1  (2.3%) | 24  (11.3%) | 10  (10.0%) | 4  (1.1%) |  |  |
| Agree | 5  (11.6%) | 66  (31.0%) | 19  (19.0%) | 9  (2.4%) |  |  |
| Strongly agree | 3  (7.0%) | 40  (18.8%) | 8  (8.0%) | 15  (4.1%) |  |  |
| No information provided | 31  (72.1%) | 81  (38.0%) | 62  (62.0%) | 338  (91.8%) |  |  |
| **The employees at this practice work well as a team. (B2.21)** |  |  |  |  | **<0.001** | 0.141 |
| Strongly disagree | 0  (0.0%) | 2  (0.9%) | 0  (0.0%) | 0  (0.0%) |  |  |
| Disagree | 0  (0.0%) | 2  (0.9%) | 1  (1.0%) | 0  (0.0%) |  |  |
| Neither agree nor disagree | 4  (9.3%) | 7  (3.3%) | 2  (2.0%) | 3  (0.8%) |  |  |
| Agree | 2  (4.7%) | 51  (23.0%) | 16  (16.0%) | 9  (2.4%) |  |  |
| Strongly agree | 6  (14.0%) | 70  (32.9%) | 19  (19.0%) | 18  (4.9%) |  |  |
| No information provided | 31  (72.1%) | 81  (38.0%) | 62  (62.0%) | 338  (91.8%) |  |  |
| **It** **seems that most of the employees at this practice enjoy their work. (B1.22)** |  |  |  |  | 0.354 | 0.507 |
| Strongly disagree | 0  (0.0%) | 1  (0.5%) | 0  (0.0%) | 0  (0.0%) |  |  |
| Disagree | 0  (0.0%) | 3  (1.4%) | 0  (0.0%) | 0  (0.0%) |  |  |
| Neither agree nor disagree | 2  (4.7%) | 8  (3.8%) | 2  (2.0%) | 1  (0.3%) |  |  |
| Agree | 8  (18.6%) | 64  (30.0%) | 18  (18.0%) | 10  (2.7%) |  |  |
| Strongly agree | 2  (4.7%) | 56  (26.3%) | 18  (18.0%) | 19  (5.2%) |  |  |
| No information provided | 31  (72.1%) | 81  (38.0%) | 62  (62.0%) | 338  (91.8%) |  |  |
| **This practice is characterised by positive cooperation and a positive working atmosphere. (B2.23)** |  |  |  |  | 0.220 | 0.112 |
| Strongly disagree | 0  (0.0%) | 2  (0.9%) | 0  (0.0%) | 0  (0.0%) |  |  |
| Disagree | 0  (0.0%) | 1  (0.5%) | 1  (1.0%) | 0  (0.0%) |  |  |
| Neither agree nor disagree | 3  (7.0%) | 8  (3.8%) | 1  (1.0%) | 2  (0.5%) |  |  |
| Agree | 7  (16.3%) | 55  (25.8%) | 15  (15.0%) | 10  (2.7%) |  |  |
| Strongly agree | 2  (4.7%) | 65  (30.5%) | 21  (21.0%) | 18  (4.9%) |  |  |
| No information provided | 31  (72.1%) | 82  (38.5%) | 62  (62.0%) | 338  (91.8%) |  |  |
| **PAR measure** | 4.10  (0.34) | 4.12  (0.49) | 4.12  (0.39) | 4.26  (0.39) | 0.491 | 0.985 |
| **C: Introducing (implementing) AdAM software into your daily practice / Organisational readiness for implementing change (ORIC) measure** |  |  |  |  |  |  |
| **The people working in this practice demonstrate a high level of commitment to implementing the content of the AdAM project, i.e. using the software and dealing with any consequences for patient care that arise. (C1.1)** |  |  |  |  | **<0.001** | **<0.001** |
| Strongly disagree | 1  (2.3%) | 9  (4.2%) | 3  (3.0%) | 4  (1.1%) |  |  |
| Disagree | 1  (2.3%) | 25  (11.7%) | 18  (18.0%) | 4  (1.1%) |  |  |
| Neither agree nor disagree | 6  (14.0%) | 43  (20.2%) | 9  (9.0%) | 9  (2.4%) |  |  |
| Agree | 2  (4.7%) | 38  (17.8%) | 5  (5.0%) | 2  (0.5%) |  |  |
| Strongly agree | 2  (4.7%) | 16  (7.5%) | 2  (2.0%) | 5  (1.4%) |  |  |
| No information provided | 31  (72.1%) | 82  (38.5%) | 63  (63.0%) | 344  (93.5%) |  |  |
| **The people working in this practice will do whatever is necessary to implement the content of the AdAM project. (C1.2)** |  |  |  |  | **<0.001** | **<0.001** |
| Strongly disagree | 1  (2.3%) | 8  (3.8%) | 5  (5.0%) | 4  (1.1%) |  |  |
| Disagree | 1  (2.3%) | 22  (10.3%) | 13  (13.0%) | 4  (1.1%) |  |  |
| Neither agree nor disagree | 3  (7.0%) | 33  (15.5%) | 10  (10.0%) | 11  (3.0%) |  |  |
| Agree | 5  (11.6%) | 49  (23.0%) | 8  (8.0%) | 1  (0.3%) |  |  |
| Strongly agree | 2  (4.7%) | 18  (8.5%) | 1  (1.0%) | 6  (1.6%) |  |  |
| No information provided | 31  (72.1%) | 83  (39.0%) | 63  (63.0%) | 342  (92.9%) |  |  |
| **The people who work at this practice want to implement the content of the AdAM project. (C1.3)** |  |  |  |  | **<0.001** | **<0.001** |
| Strongly disagree | 1  (2.3%) | 7  (3.3%) | 4  (4.0%) | 5  (1.4%) |  |  |
| Disagree | 0  (0.0%) | 22  (10.3%) | 7  (7.0%) | 1  (0.3%) |  |  |
| Neither agree nor disagree | 3  (7.0%) | 36  (16.9%) | 16  (16.0%) | 8  (2.2%) |  |  |
| Agree | 6  (14.0%) | 47  (22.1%) | 9  (9.0%) | 7  (1.9%) |  |  |
| Strongly agree | 2  (4.7%) | 18  (8.5%) | 1  (1.0%) | 5  (1.4%) |  |  |
| No information provided | 31  (72.1%) | 83  (39.0%) | 63  (63.0%) | 342  (92.9%) |  |  |
| **The people working at this practice are determined to implement the content of the AdAM project. (C1.4)** |  |  |  |  | **<0.001** | **<0.001** |
| Strongly disagree | 1  (2.3%) | 6  (2.8%) | 5  (5.0%) | 5  (1.4%) |  |  |
| Disagree | 2  (4.7%) | 26  (12.2%) | 12  (12.0%) | 3  (0.8%) |  |  |
| Neither agree nor disagree | 2  (4.7%) | 38  (17.8%) | 10  (10.0%) | 10  (2.7%) |  |  |
| Agree | 4  (9.3%) | 43  (20.2%) | 10  (10.0%) | 3  (0.8%) |  |  |
| Strongly agree | 3  (7.0%) | 17  (8.0%) | 0  (0.0%) | 5  (1.4%) |  |  |
| No information provided | 31  (72.1%) | 83  (39.0%) | 63  (63.0%) | 342  (92.9%) |  |  |
| **The people working in this practice are motivated to implement the content of the AdAM project. (C1.5)** |  |  |  |  | **<0.001** | **<0.001** |
| Strongly disagree | 1  (2.3%) | 5  (2.3%) | 6  (6.0%) | 5  (1.4%) |  |  |
| Disagree | 0  (0.0%) | 21  (9.9%) | 6  (6.0%) | 1  (0.3%) |  |  |
| Neither agree nor disagree | 1  (2.3%) | 29  (13.6%) | 11  (11.0%) | 7  (1.9%) |  |  |
| Agree | 6  (14.0%) | 56  (26.3%) | 13  (13.0%) | 8  (2.2%) |  |  |
| Strongly agree | 4  (0.9%) | 20  (9.4%) | 1  (1.0%) | 6  (1.6%) |  |  |
| No information provided | 31  (72.1%) | 82  (38.5%) | 63  (63.0%) | 341  (92.7%) |  |  |
| **Challenges may arise during the implementation of the AdAM project. However, the people working here are confident that they will be able to overcome these challenges. (C1.6)** |  |  |  |  | **<0.001** | 0.181 |
| Strongly disagree | 0  (0.0%) | 5  (2.3%) | 4  (4.0%) | 4  (1.1%) |  |  |
| Disagree | 2  (4.7%) | 13  (6.1%) | 7  (7.0%) | 1  (0.3%) |  |  |
| Neither agree nor disagree | 2  (4.7%) | 33  (15.5%) | 12  (12.0%) | 8  (2.2%) |  |  |
| Agree | 5  (11.6%) | 63  (29.6%) | 11  (11.0%) | 7  (1.9%) |  |  |
| Strongly agree | 3  (7.0%) | 17  (8.0%) | 3  (3.0%) | 6  (1.6%) |  |  |
| No information provided | 31  (72.1%) | 82  (38.5%) | 63  (63.0%) | 342  (92.9%) |  |  |
| **The people working at this practice are confident that they can oversee the implementation of the AdAM project. (C1.7)** |  |  |  |  | **<0.001** | 0.203 |
| Strongly disagree | 1  (2.3%) | 7  (3.3%) | 4  (4.0%) | 5  c |  |  |
| Disagree | 0  (0.0%) | 20  (9.4%) | 4  (4.0%) | 1  (0.3%) |  |  |
| Neither agree nor disagree | 3  (7.0%) | 32  (15.0%) | 16  (16.0%) | 9  (2.4%) |  |  |
| Agree | 6  (14.0%) | 58  (27.2%) | 11  (11.0%) | 4  (1.1%) |  |  |
| Strongly agree | 2  (4.7%) | 14  (6.6%) | 2  (2.0%) | 7  (1.9%) |  |  |
| No information provided | 31  (72.1%) | 82  (38.5%) | 63  (63.0%) | 342  (92.9%) |  |  |
| **The people who work at this practice are confident that they can organise tasks in such a way that they are implemented smoothly. (C1.8)** |  |  |  |  | **<0.001** | 0.083 |
| Strongly disagree | 0  (0.0%) | 3  (1.4%) | 4  (4.0%) | 4  (1.1%) |  |  |
| Disagree | 1  (2.3%) | 14  (6.6%) | 5  (5.0%) | 3  (0.8%) |  |  |
| Neither agree nor disagree | 2  (4.7%) | 32  (15.0%) | 15  (15.0%) | 6  (1.6%) |  |  |
| Agree | 7  (16.3%) | 61  (28.6%) | 11  (11.0%) | 7  (1.9%) |  |  |
| Strongly agree | 2  (4.7%) | 20  (9.4%) | 2  (2.0%) | 7  (1.9%) |  |  |
| No information provided | 31  (72.1%) | 83  (39.0%) | 63  (63.0%) | 341  (92.7%) |  |  |
| **The people who work here are confident that the practice will support them in implementing the content of the AdAM project. (C1.9)** |  |  |  |  | **<0.001** | **<0.001** |
| Strongly disagree | 0  (0.0%) | 3  (1.4%) | 5  (5.0%) | 4  (1.1%) |  |  |
| Disagree | 1  (2.3%) | 19  (8.9%) | 6  (6.0%) | 3  (0.8%) |  |  |
| Neither agree nor disagree | 4  (9.3%) | 29  (13.6%) | 15  (15.0%) | 8  (2.2%) |  |  |
| Agree | 6  (14.0%) | 63  (29.6%) | 9  (9.0%) | 6  (1.6%) |  |  |
| Strongly agree | 1  (2.3%) | 17  (8.0%) | 2  (2.0%) | 6  (1.6%) |  |  |
| No information provided | 31  (72.1%) | 82  (38.5%) | 63  (63.0%) | 341  (92.7%) |  |  |
| **ORIC measure*** | 3.64  (0.92) | 3.43  (0.93) | 2.87  (0.91) | 3.18  (1.33) | **0.013** | **0.003** |
| **ORIC (change commitment; item 1-5)** | 3.58  (1.08) | 3.35  (1.01) | 2.75  (0.95) | 3.08  (1.34) | **0.011** | **0.003** |
| **ORIC (change efficacy; item 6-9)*** | 3.71  (0.78) | 3.53  (0.88) | 3.03  (0.96) | 3.35  (1.33) | **0.029** | **0.007** |
| **D: Using AdAM software in your daily practice / German Normalisation Process Theory (G-NoMAD) measure** |  |  |  |  |  |  |
| **We are comfortable using the AdAM software. (D1.1.1)** |  |  |  |  | **<0.001** | **<0.001** |
| 1 (Not at all) | 0  (0.0%) | 1  (0.5%) | 3  (3.0%) | 8  (2.2%) |  |  |
| 2 | 0  (0.0%) | 2  (0.9%) | 3  (3.0%) | 3  (0.8%) |  |  |
| 3 | 0  (0.0%) | 5  (2.3%) | 3  (3.0%) | 3  (0.8%) |  |  |
| 4 | 0  (0.0%) | 8  (3.8%) | 3  (3.0%) | 1  (0.3%) |  |  |
| 5 (Neither) | 0  (0.0%) | 6  (2.8%) | 3  (3.0%) | 2  (0.5%) |  |  |
| 6 | 2  (4.7%) | 11  (5.2%) | 1  (1.0%) | 4  (1.1%) |  |  |
| 7 | 2  (4.7%) | 28  (13.1%) | 7  (7.0%) | 2  (0.5%) |  |  |
| 8 | 5  (11.6%) | 42  (19.7%) | 7  (7.0%) | 4  (1.1%) |  |  |
| 9 | 3  (7.0%) | 16  (7.5%) | 5  (5.0%) | 2  (0.5%) |  |  |
| 10 (Completely) | 0  (0.0%) | 13  (6.1%) | 3  (3.0%) | 1  (0.3%) |  |  |
| No information provided | 31  (72.1%) | 81  (38.0%) | 62  (62.0%) | 338  (91.8%) |  |  |
| **Using AdAM software has become an integral part of our work routine. (D1.1.2)** |  |  |  |  | **<0.001** | **<0.001** |
| 1 (Not at all) | 1  (2.3%) | 14  (6.6%) | 8  (8.0%) | 14  (3.8%) |  |  |
| 2 | 2  (4.7%) | 10  (4.7%) | 6  (6.0%) | 4  (1.1%) |  |  |
| 3 | 1  (2.3%) | 18  (8.5%) | 5  (5.0%) | 4  (1.1%) |  |  |
| 4 | 2  (4.7%) | 14  (6.6%) | 6  (6.0%) | 0  (0.0%) |  |  |
| 5 (Neither) | 1  (2.3%) | 13  (6.1%) | 3  (3.0%) | 1  (0.3%) |  |  |
| 6 | 3  (7.0%) | 24  (11.3%) | 4  (4.0%) | 2  (0.5%) |  |  |
| 7 | 1  (2.3%) | 17  (8.0%) | 3  (3.0%) | 2  (0.5%) |  |  |
| 8 | 1  (2.3%) | 14  (6.6%) | 1  (1.0%) | 3  (0.8%) |  |  |
| 9 | 0  (0.0%) | 4  (1.9%) | 1  (1.0%) | 0  (0.0%) |  |  |
| 10 (Completely) | 0  (0.0%) | 4  (1.9%) | 1  (1.0%) | 0  (0.0%) |  |  |
| No information provided | 31  (72.1%) | 81  (38.0%) | 62  (62.0%) | 338  (91.8%) |  |  |
| **The use of AdAM software will become/remain an integral part of our work routine in the future. (D1.1.3)** |  |  |  |  | **<0.001** | **<0.001** |
| 1 (Not at all) | 2  (4.7%) | 11  (5.2%) | 5  (5.0%) | 9  (2.4%) |  |  |
| 2 | 1  (2.3%) | 14  (6.6%) | 3  (3.0%) | 3  (0.8%) |  |  |
| 3 | 1  (2.3%) | 8  (3.8%) | 8  (8.0%) | 3  (0.8%) |  |  |
| 4 | 1  (2.3%) | 8  (3.8%) | 5  (5.0%) | 1  (0.3%) |  |  |
| 5 (Neither) | 1  (2.3%) | 15  (7.0%) | 3  (3.0%) | 0  (0.0%) |  |  |
| 6 | 3  (7.0%) | 20  (9.4%) | 4  (4.0%) | 4  (1.1%) |  |  |
| 7 | 1  (2.3%) | 19  (8.9%) | 4  (4.0%) | 2  (0.5%) |  |  |
| 8 | 2  (4.7%) | 26  (12.2%) | 4  (4.0%) | 2  (0.5%) |  |  |
| 9 | 0  (0.0%) | 5  (2.3%) | 0  (0.0%) | 2  (0.5%) |  |  |
| 10 (Completely) | 0  (0.0%) | 5  (2.3%) | 2  (2.0%) | 2  (0.5%) |  |  |
| No information provided | 31  (72.1%) | 82  (38.5%) | 62  (62.0%) | 340  (92.4%) |  |  |
| **I can understand how medication management with the AdAM software differs from our previous medication management system. (D1.2.1)** |  |  |  |  | **<0.001** | 0.777 |
| Strongly disagree | 0  (0.0%) | 6  (2.8%) | 3  (3.0%) | 5  (1.4%) |  |  |
| Disagree | 1  (2.3%) | 24  (11.3%) | 8  (8.0%) | 4  (1.1%) |  |  |
| Neither agree nor disagree | 3  (7.0%) | 34  (16.0%) | 11  (11.0%) | 5  (1.4%) |  |  |
| Agree | 7  (16.3%) | 51  (23.9%) | 10  (10.0%) | 5  (1.4%) |  |  |
| Strongly agree | 1  (2.3%) | 17  (8.0%) | 6  (6.0%) | 7  (1.9%) |  |  |
| No information provided | 31  (72.1%) | 81  (38.0%) | 62  (62.0%) | 342  (92.9%) |  |  |
| **The employees of this practice have a common understanding of the purpose of the AdAM software. (D1.2.2)** |  |  |  |  | **<0.001** | 0.321 |
| Strongly disagree | 1  (2.3%) | 11  (5.2%) | 6  (6.0%) | 4  (1.1%) |  |  |
| Disagree | 2  (4.7%) | 21  (9.9%) | 10  (!0.0%) | 4  (1.1%) |  |  |
| Neither agree nor disagree | 5  (11.6%) | 35  (16.4%) | 12  (12.0%) | 5  (1.4%) |  |  |
| Agree | 4  (9.3%) | 51  (23.9%) | 8  (8.0%) | 7  (1.9%) |  |  |
| Strongly agree | 0  (0.0%) | 14  (6.6%) | 2  (2.0%) | 5  (1.4%) |  |  |
| No information provided | 31  (72.1%) | 81  (38.0%) | 62  (62.0%) | 343  (93.2%) |  |  |
| **I understand the effect that using AdAM software has on my own work.** |  |  |  |  | **<0.001** | **<0.001** |
| Strongly disagree | 1  (2.3%) | 4  (1.9%) | 3  (3.0%) | 4  (1.1%) |  |  |
| Disagree | 1  (2.3%) | 16  (7.5%) | 9  (9.0%) | 3  (0.8%) |  |  |
| Neither agree nor disagree | 1  (2.3%) | 33  (15.5%) | 13  (13.0%) | 5  (1.4%) |  |  |
| Agree | 7  (16.3%) | 57  (26.8%) | 9  (9.0%) | 7  (1.9%) |  |  |
| Strongly agree | 2  (4.7%) | 22  (10.3%) | 4  (4.0%) | 6  (1.6%) |  |  |
| No information provided | 31  (72.1%) | 81  (38.0%) | 62  (62.0%) | 343  (93.2%) |  |  |
| **I recognise the potential value of the AdAM software in relation to my work. (D1.2.3)** |  |  |  |  | **<0.001** | 0.102 |
| Strongly disagree | 1  (2.3%) | 4  (1.9%) | 2  (2.0%) | 5  (1.4%) |  |  |
| Disagree | 1  (2.3%) | 7  (3.3%) | 8  (8.0%) | 2  (0.5%) |  |  |
| Neither agree nor disagree | 2  (4.7%) | 34  (16.0%) | 8  (8.0%) | 6  (1.6%) |  |  |
| Agree | 4  (9.3%) | 56  (26.3%) | 15  (15.0%) | 6  (1.6%) |  |  |
| Strongly agree | 4  (9.3%) | 32  (15.0%) | 5  (5.0%) | 6  (1.6%) |  |  |
| No information provided | 31  (72.1%) | 80  (37.6%) | 62  (62.0%) | 343  (93.2%) |  |  |
| **Key individuals promote the use of AdAM software and encourage others to join them. (D1.2.4)** |  |  |  |  | **<0.001** | **<0.001** |
| Strongly disagree | 1  (2.3%) | 14  (6.6%) | 7  (7.0%) | 7  (1.9%) |  |  |
| Disagree | 1  (2.3%) | 25  (11.7%) | 10  (10.0%) | 4  (1.1%) |  |  |
| Neither agree nor disagree | 3  (7.0%) | 24  (11.3%) | 12  (12.0%) | 2  (0.5%) |  |  |
| Agree | 4  (9.3%) | 48  (22.5%) | 5  (5.0%) | 4  (1.1%) |  |  |
| Strongly agree | 2  (4.7%) | 21  (10.0%) | 4  (4.0%) | 8  (2.2%) |  |  |
| No information provided | 32  (74.4%) | 81  (38.0%) | 62  (62.0%) | 343  (93.2%) |  |  |
| **G-NoMAD Subscale Coherence** | 3.52  (0.90) | 3.50  (0.86) | 3.09  (0.98) | 3.24  (1.32) | 0.091 | **0.038** |
| **I believe that my role requires me to be involved in using the AdAM software. (D1.2.5)** |  |  |  |  | **<0.001** | **0.024** |
| Strongly disagree | 1  (2.3%) | 9  (4.2%) | 1  (1.0%) | 3  (0.8%) |  |  |
| Disagree | 1  (2.3%) | 5  (2.3%) | 6  (6.0%) | 3  (0.8%) |  |  |
| Neither agree nor disagree | 1  (2.3%) | 14  (6.6%) | 10  (10.0%) | 3  (0.8%) |  |  |
| Agree | 5  (11.6%) | 70  (32.9%) | 15  (15.0%) | 7  (1.9%) |  |  |
| Strongly agree | 4  (9.3%) | 34  (16.0%) | 5  (5.0%) | 9  (2.4%) |  |  |
| No information provided | 31  (72.1%) | 81  (38.0%) | 63  (63.0%) | 343  (93.2%) |  |  |
| **I am open to exploring new ways of working with colleagues using the AdAM software. (D1.2.6)** |  |  |  |  | **<0.001** | 0.284 |
| Strongly disagree | 1  (2.3%) | 10  (4.7%) | 4  (4.0%) | 3  (0.8%) |  |  |
| Disagree | 2  (4.7%) | 11  (5.2%) | 4  (4.0%) | 3  (0.8%) |  |  |
| Neither agree nor disagree | 0  (0.0%) | 26  (12.2%) | 12  (!2.0%) | 4  (1.1%) |  |  |
| Agree | 7  (16.3%) | 65  (30.5%) | 13  (13.0%) | 9  (2.4%) |  |  |
| Strongly agree | 2  (4.7%) | 19  (8.9%) | 4  (4.0%) | 6  (1.6%) |  |  |
| No information provided | 31  (72.1%) | 82  (38.5%) | 63  (63.0%) | 343  (93.2%) |  |  |
| **I will continue to manage medication using the AdAM software. (D1.2.7)** |  |  |  |  | **<0.001** | **<0.001** |
| Strongly disagree | 1  (2.3%) | 7  (3.3%) | 3  (3.0%) | 6  (1.6%) |  |  |
| Disagree | 1  (2.3%) | 19  (8.9%) | 10  (10.0%) | 2  (0.5%) |  |  |
| Neither agree nor disagree | 1  (2.3%) | 23  (10.8%) | 9  (9.0%) | 4  (1.1%) |  |  |
| Agree | 6  (14.0%) | 50  (23.5%) | 14  (14.0%) | 6  (1.6%) |  |  |
| Strongly agree | 3  (7.0%) | 33  (15.5%) | 1  (1.0%) | 7  (1.9%) |  |  |
| No information provided | 31  (72.1%) | 81  (38.0%) | 63  (63.0%) | 343  (93.2%) |  |  |
| **Integrating the AdAM software into my existing work would be straightforward. (D1.2.8)** |  |  |  |  | **<0.001** | 0.871 |
| Strongly disagree | 2  (4.7%) | 19  (8.9%) | 7  (7.0%) | 8  (2.2%) |  |  |
| Disagree | 2  (4.7%) | 47  (22.1%) | 14  (14.0%) | 6  (1.6%) |  |  |
| Neither agree nor disagree | 5  (11.6%) | 37  (17.4%) | 10  (10.0%) | 3  (0.8%) |  |  |
| Agree | 2  (4.7%) | 22  (10.3%) | 5  (5.0%) | 4  (1.1%) |  |  |
| Strongly agree | 1  (2.3%) | 8  (3.8%) | 1  (1.0%) | 4  (1.1%) |  |  |
| No information provided | 31  (72.1%) | 80  (37.6%) | 63  (63.0%) | 343  (93.2%) |  |  |
| **G-NoMAD Subscale Cognitive Participation** | 3.80  (0.94) | 3.60  (0.91) | 3.11  (0.86) | 3.36  (1.34) | **0.033** | **0.009** |
| **Using AdAM software has a negative impact on working relationships. (D1.2.9)** |  |  |  |  | **<0.001** | 0.099 |
| Strongly disagree | 2  (4.7%) | 62  (29.1%) | 13  (13.0%) | 10  (2.7%) |  |  |
| Disagree | 7  (16.3%) | 43  (20.2%) | 16  (16.0%) | 6  (1.6%) |  |  |
| Neither agree nor disagree | 1  (2.3%) | 16  (7.5%) | 5  (5.0%) | 3  (0.8%) |  |  |
| Agree | 0  (0.0%) | 8  (3.8%) | 3  (3.0%) | 2  (0.5%) |  |  |
| Strongly agree | 1  (2.3%) | 0  (0.0%) | 0  (0.0%) | 1  (0.3%) |  |  |
| No information provided | 32  (74.4%) | 84  (39.4%) | 63  (63.0%) | 346  (94.0%) |  |  |
| **I am confident that others will be able to use the AdAM software. (D1.2.10)** |  |  |  |  | **<0.001** | 0.480 |
| Strongly disagree | 1  (2.3%) | 6  (2.8%) | 4  (4.0%) | 1  (0.3%) |  |  |
| Disagree | 2  (4.7%) | 15  (7.0%) | 1  (1.0%) | 1  (0.3%) |  |  |
| Neither agree nor disagree | 4  (9.3%) | 42  (19.7%) | 12  (12.0%) | 4  (1.1%) |  |  |
| Agree | 4  (9.3%) | 56  (26.3%) | 16  (16.0%) | 8  (2.2%) |  |  |
| Strongly agree | 0  (0.0%) | 9  (4.2%) | 4  (4.0%) | 6  (1.6%) |  |  |
| No information provided | 32  (74.4%) | 85  (39.9%) | 63  (63.0%) | 348  (94.6%) |  |  |
| **Employees with the appropriate skills to use the AdAM software are assigned to the work. (D1.2.11)** |  |  |  |  | **<0.001** | 0.894 |
| Strongly disagree | 2  (4.7%) | 14  (6.6%) | 4  (4.0%) | 1  (0.3%) |  |  |
| Disagree | 2  (4.7%) | 14  (6.6%) | 5  (5.0%) | 0  (0.0%) |  |  |
| Neither agree nor disagree | 2  (4.7%) | 23  (10.8%) | 8  (8.0%) | 5  (1.4%) |  |  |
| Agree | 3  (7.0%) | 58  (27.2%) | 13  (13.0%) | 8  (2.2%) |  |  |
| Strongly agree | 2  (4.7%) | 17  (8.0%) | 5  (5.0%) | 7  (1.9%) |  |  |
| No information provided | 32  (74.4%) | 87  (40.8%) | 65  (65.0%) | 347  (94.3%) |  |  |
| **Staff are provided with sufficient training to enable them to use the AdAM software. (D1.2.12)** |  |  |  |  | **<0.001** | 0.119 |
| Strongly disagree | 3  (7.0%) | 21  (9.9%) | 6  (6.0%) | 5  (1.4%) |  |  |
| Disagree | 1  (2.3%) | 28  (13.1%) | 15  (15.0%) | 3  (0.8%) |  |  |
| Neither agree nor disagree | 4  (9.3%) | 50  (23.5%) | 7  (7.0%) | 5  (1.4%) |  |  |
| Agree | 2  (4.7%) | 24  (11.3%) | 5  (5.0%) | 5  (1.4%) |  |  |
| Strongly agree | 0  (0.0%) | 4  (1.9%) | 3  (3.0%) | 3  (0.8%) |  |  |
| No information provided | 33  (76.7%) | 86  (40.4%) | 64  (64.0%) | 347  (94.3%) |  |  |
| **There are sufficient resources available to use the AdAM software. (D1.2.13)** |  |  |  |  | **<0.001** | 0.150 |
| Strongly disagree | 1  (2.3%) | 16  (7.5%) | 8  (8.0%) | 6  (1.6%) |  |  |
| Disagree | 0  (0.0%) | 38  (17.8%) | 13  (13.0%) | 7  (1.9%) |  |  |
| Neither agree nor disagree | 6  (14.0%) | 42  (19.7%) | 10  (10.0%) | 2  (0.5%) |  |  |
| Agree | 2  (4.7%) | 29  (13.6%) | 5  (5.0%) | 5  (1.4%) |  |  |
| Strongly agree | 1  (2.3%) | 4  (1.9%) | 1  (1.0%) | 4  (1.1%) |  |  |
| No information provided | 33  (76.7%) | 84  (39.4%) | 63  (63.0%) | 344  (93.5%) |  |  |
| **I support the AdAM project as required. (D1.2.14)** |  |  |  |  | **<0.001** | **<0.001** |
| Strongly disagree | 1  (2.3%) | 3  (1.4%) | 4  (4.0%) | 4  (1.1%) |  |  |
| Disagree | 2  (4.7%) | 12  (5.6%) | 14  (14.0%) | 3  (0.8%) |  |  |
| Neither agree nor disagree | 2  (4.7%) | 37  (17.4%) | 5  (5.0%) | 8  (2.2%) |  |  |
| Agree | 5  (11.6%) | 62  (29.1%) | 11  (11.0%) | 5  (1.4%) |  |  |
| Strongly agree | 1  (2.3%) | 14  (6.6%) | 3  (3.0%) | 5  (1.4%) |  |  |
| No information provided | 32  (74.4%) | 85  (39.9%) | 63  (63.0%) | 343  (93.2%) |  |  |
| **I am familiar with reports on the effects of the AdAM software. (D1.2.15)** |  |  |  |  | **<0.001** | 0.527 |
| Strongly disagree | 2  (4.7%) | 33  (15.5%) | 10  (10.0%) | 10  (2.7%) |  |  |
| Disagree | 3  (7.0%) | 41  (19.2%) | 12  (12.0%) | 4  (1.1%) |  |  |
| Neither agree nor disagree | 6  (14.0%) | 28  (13.1%) | 6  (6.0%) | 6  (1.6%) |  |  |
| Agree | 0  (0.0%) | 21  (9.9%) | 7  (7.0%) | 3  (0.8%) |  |  |
| Strongly agree | 0  (0.0%) | 6  (2.8%) | 2  (2.0%) | 1  (0.3%) |  |  |
| No information provided | 32  (74.4%) | 84  (39.4%) | 63  (63.0%) | 344  (93.5%) |  |  |
| **G-NoMAD Subscale Collective Action** | 2.91  (0.60) | 2.89  (0.60) | 2.73  (0.67) | 3.04  (0.80) | 0.383 | 0.401 |
| **Employees agree that the AdAM software is worthwhile. (D1.2.16)** |  |  |  |  | **<0.001** | **<0.001** |
| Strongly disagree | 2  (4.7%) | 14  (6.6%) | 5  (5.0%) | 6  (1.6%) |  |  |
| Disagree | 2  (4.7%) | 27  (12.7%) | 15  (15.0%) | 4  (1.1%) |  |  |
| Neither agree nor disagree | 2  (4.7%) | 46  (21.6%) | 11  (11.0%) | 7  (1.9%) |  |  |
| Agree | 5  (11.6%) | 29  (13.6%) | 5  (5.0%) | 2  (0.5%) |  |  |
| Strongly agree | 0  (0.0%) | 11  (5.2%) | 1  (1.0%) | 3  (0.8%) |  |  |
| No information provided | 32  (74.4%) | 86  (40.4%) | 63  (63.0%) | 346  (94.0%) |  |  |
| **I appreciate the impact that the AdAM software has on my work. (D1.2.17)** |  |  |  |  | **<0.001** | 0.350 |
| Strongly disagree | 1  (2.3%) | 9  (4.2%) | 3  (3.0%) | 5  (1.4%) |  |  |
| Disagree | 1  (2.3%) | 20  (9.4%) | 10  (10.0%) | 4  (1.1%) |  |  |
| Neither agree nor disagree | 1  (2.3%) | 33  (15.5%) | 11  (11.0%) | 7  (1.9%) |  |  |
| Agree | 7  (16.3%) | 48  (22.5%) | 11  (11.0%) | 3  (0.8%) |  |  |
| Strongly agree | 1  (2.3%) | 19  (8.9%) | 2  (2.0%) | 4  (1.1%) |  |  |
| No information provided | 32  (74.4%) | 84  (39.4%) | 63  (63.0%) | 345  (93.8%) |  |  |
| **Any feedback on the AdAM software can be used to make improvements in the future. (D1.2.18)** |  |  |  |  | **<0.001** | 0.613 |
| Strongly disagree | 0  (0.0%) | 4  (1.9%) | 2  (2.0%) | 3  (0.8%) |  |  |
| Disagree | 1  (2.3%) | 9  (4.2%) | 2  (2.0%) | 2  (0.5%) |  |  |
| Neither agree nor disagree | 1  (2.3%) | 24  (11.3%) | 8  (8.0%) | 1  (0.3%) |  |  |
| Agree | 7  (16.3%) | 62  (29.1%) | 21  (21.0%) | 9  (2.4%) |  |  |
| Strongly agree | 2  (4.7%) | 29  (4.2%) | 3  (3.0%) | 8  (2.2%) |  |  |
| No information provided | 32  (74.4%) | 85  (39.9%) | 64  (64.0%) | 345  (93.8%) |  |  |
| **I can customise my approach to working with the AdAM software. (D1.2.19)** |  |  |  |  | **<0.001** | 0.800 |
| Strongly disagree | 1  (2.3%) | 7  (3.3%) | 5  (5.0%) | 3  (0.8%) |  |  |
| Disagree | 1  (2.3%) | 23  (10.8%) | 7  (7.0%) | 5  (1.4%) |  |  |
| Neither agree nor disagree | 3  (7.0%) | 41  (19.2%) | 11  (11.0%) | 4  (1.1%) |  |  |
| Agree | 5  (11.6%) | 45  (21.1%) | 11  (11.0%) | 5  (1.4%) |  |  |
| Strongly agree | 1  (2.3%) | 12  (5.6%) | 2  (2.0%) | 5  (1.4%) |  |  |
| No information provided | 32  (74.4%) | 85  (39.9%) | 64  (64.0%) | 346  (94.0%) |  |  |
| **G-NoMAD Subscale Reflexive Monitoring** | 3.22  (0.81) | 3.17  (0.85) | 2.90  (0.87) | 2.97  (1.18) | 0.376 | 0.236 |
| **G-NoMAD Total** | 3.43  (0.49) | 3.23  (0.69) | 2.96  (0.71) | 3.16  (0.97) | 0.178 | 0.066 |
| **E: Summary** |  |  |  |  |  |  |
| **We have the time and resources necessary to devote ourselves appropriately to such a project. (E1.1)** |  |  |  |  | **<0.001** | 0.611 |
| Strongly disagree | 1  (2.3%) | 14  (6.6%) | 5  (5.0%) | 6  (1.6%) |  |  |
| Disagree | 4  (9.3%) | 42  (19.7%) | 19  (19.0%) | 8  (2.2%) |  |  |
| Neither agree nor disagree | 5  (11.6%) | 48  (22.5%) | 10  (10.0%) | 6  (1.6%) |  |  |
| Agree | 2  (4.7%) | 26  (12.2%) | 4  (4.0%) | 1  (0.3%) |  |  |
| Strongly agree | 0  (0.0%) | 3  (1.4%) | 0  (0.0%) | 5  (1.4%) |  |  |
| No information provided | 31  (72.1%) | 80  (37.6%) | 62  (62.0%) | 342  (92.9%) |  |  |
| **We have the human resources necessary to devote ourselves appropriately to such a project. (E1.2)** |  |  |  |  | **<0.001** | 0.292 |
| Strongly disagree | 1  (2.3%) | 8  (3.8%) | 3  (3.0%) | 6  (1.6%) |  |  |
| Disagree | 3  (7.0%) | 30  (14.1%) | 13  (13.0%) | 6  (1.6%) |  |  |
| Neither agree nor disagree | 2  (4.7%) | 50  (23.5%) | 15  (15.0%) | 6  (1.6%) |  |  |
| Agree | 6  (14.0%) | 37  (17.4%) | 7  (7.0%) | 1  (0.3%) |  |  |
| Strongly agree | 0  (0.0%) | 8  (3.8%) | 0  (0.0%) | 7  (1.9%) |  |  |
| No information provided | 31  (72.1%) | 80  (37.6%) | 62  (62.0%) | 342  (92.9%) |  |  |
| **We consider AdAM software an enhancement to our existing technical equipment. (E1.3)** |  |  |  |  | **<0.001** | **<0.001** |
| Strongly disagree | 2  (4.7%) | 12  (5.6%) | 7  (7.0%) | 7  (1.9%) |  |  |
| Disagree | 1  (2.3%) | 23  (10.8%) | 15  (15.0%) | 2  (0.5%) |  |  |
| Neither agree nor disagree | 1  (2.3%) | 40  (18.8%) | 8  (8.0%) | 4  (1.1%) |  |  |
| Agree | 6  (14.0%) | 46  (21.6%) | 7  (7.0%) | 6  (1.6%) |  |  |
| Strongly agree | 2  (4.7%) | 12  (5.6%) | 1  (1.0%) | 4  (1.1%) |  |  |
| No information provided | 31  (72.1%) | 80  (37.6%) | 62  (62.0%) | 345  (93.8%) |  |  |
| **The billing data provided by BARMER is valuable for treating my patients. (E1.4)** |  |  |  |  | **<0.001** | 0.919 |
| Strongly disagree | 0  (0.0%) | 8  (3.8%) | 4  (4.0%) | 4  (1.1%) |  |  |
| Disagree | 1  (2.3%) | 21  (9.9%) | 7  (7.0%) | 2  (0.5%) |  |  |
| Neither agree nor disagree | 3  (7.0%) | 32  (15.0%) | 10  (10.0%) | 5  (1.4%) |  |  |
| Agree | 5  (11.6%) | 53  (24.9%) | 13  (13.0%) | 5  (1.4%) |  |  |
| Strongly agree | 2  (4.7%) | 18  (8.5%) | 3  (3.0%) | 7  (1.9%) |  |  |
| No information provided | 32  (74.4%) | 81  (38.0%) | 63  (63.0%) | 345  (93.8%) |  |  |
| **Specialist consultations help me treat my patients. (E1.5)** |  |  |  |  | **<0.001** | 0.847 |
| Strongly disagree | 2  (4.7%) | 30  (14.1%) | 6  (6.0%) | 9  (2.4%) |  |  |
| Disagree | 5  (11.6%) | 49  (23.0%) | 13  (13.0%) | 1  (0.3%) |  |  |
| Neither agree nor disagree | 4  (9.3%) | 35  (16.4%) | 9  (9.0%) | 5  (1.4%) |  |  |
| Agree | 1  (2.3%) | 13  (6.1%) | 7  (7.0%) | 4  (1.1%) |  |  |
| Strongly agree | 0  (0.0%) | 5  (2.3%) | 0  (0.0%) | 2  (0.5%) |  |  |
| No information provided | 31  (72.1%) | 81  (38.0%) | 65  (65.0%) | 347  (94.3%) |  |  |
| **In my opinion, the project would have been more successful if it had included a technical solution for patients, such as an AdAM app. (E1.6)** |  |  |  |  | **<0.001** | 0.720 |
| Strongly disagree | 2  (4.7%) | 14  (6.6%) | 3  (3.0%) | 4  (1.1%) |  |  |
| Disagree | 3  (7.0%) | 41  (19.2%) | 9  (9.0%) | 6  (1.6%) |  |  |
| Neither agree nor disagree | 2  (4.7%) | 35  (16.4%) | 8  (8.0%) | 5  (1.4%) |  |  |
| Agree | 4  (9.3%) | 34  (16.0%) | 13  (13.0%) | 6  (1.6%) |  |  |
| Strongly agree | 0  (0.0%) | 6  (2.8%) | 4  (4.0%) | 2  (0.5%) |  |  |
| No information provided | 32  (74.4%) | 83  (39.0%) | 63  (63.0%) | 345  (93.8%) |  |  |
| **My expectations for using the AdAM software have been met. (E1.7)** |  |  |  |  | **<0.001** | 0.425 |
| Strongly disagree | 1  (2.3%) | 11  (5.2%) | 4  (4.0%) | 5  (1.4%) |  |  |
| Disagree | 1  (2.3%) | 24  (11.3%) | 13  (13.0%) | 3  (0.8%) |  |  |
| Neither agree nor disagree | 7  (16.3%) | 55  (25.8%) | 13  (13.0%) | 7  (1.9%) |  |  |
| Agree | 2  (4.7%) | 35  (16.4%) | 6  (6.0%) | 3  (0.8%) |  |  |
| Strongly agree | 0  (0.0%) | 7  (3.3%) | 2  (2.0%) | 3  (0.8%) |  |  |
| No information provided | 32  (74.4%) | 81  (38.0%) | 62  (62.0%) | 347  (94.3%) |  |  |
| **The AdAM software helps increase patient safety. (E1.8)** |  |  |  |  | **<0.001** | 0.210 |
| Strongly disagree | 1  (2.3%) | 2  (0.9%) | 1  (1.0%) | 2  (0.5%) |  |  |
| Disagree | 2  (4.7%) | 15  (7.0%) | 3  (3.0%) | 1  (0.3%) |  |  |
| Neither agree nor disagree | 0  (0.0%) | 23  (10.8%) | 10  (10.0%) | 5  (1.4%) |  |  |
| Agree | 8  (18.6%) | 70  (32.9%) | 18  (18.0%) | 8  (2.2%) |  |  |
| Strongly agree | 0  (0.0%) | 22  (10.3%) | 6  (6.0%) | 4  (1.1%) |  |  |
| No information provided | 32  (74.4%) | 81  (38.0%) | 62  (62.0%) | 348  (94.6%) |  |  |
| **I have more confidence in my decisions and actions regarding my patients' drug therapy thanks to the AdAM software. (E1.9)** |  |  |  |  | **<0.001** | 0.177 |
| Strongly disagree | 1  (2.3%) | 5  (2.3%) | 2  (2.0%) | 5  (1.4%) |  |  |
| Disagree | 1  (2.3%) | 19  (8.9%) | 8  (8.0%) | 3  (0.8%) |  |  |
| Neither agree nor disagree | 3  (7.0%) | 24  (11.3%) | 13  (13.0%) | 6  (1.6%) |  |  |
| Agree | 6  (14.0%) | 65  (30.5%) | 11  (11.0%) | 4  (1.1%) |  |  |
| Strongly agree | 0  (0.0%) | 19  (8.9%) | 4  (4.0%) | 4  (1.1%) |  |  |
| No information provided | 32  (74.4%) | 81  (38.0%) | 62  (62.0%) | 346  (94.0) |  |  |
| **I used the AdAM software for all of the enrolled patients. (E1.10)** |  |  |  |  | **<0.001** | **<0.001** |
| Strongly disagree | 1  (2.3%) | 8  (3.8%) | 9  (9.0%) | 4  (1.1%) |  |  |
| Disagree | 2  (4.7%) | 16  (7.5%) | 9  (9.0%) | 3  (0.8%) |  |  |
| Neither agree nor disagree | 3  (7.0%) | 26  (12.2%) | 4  (4.0%) | 5  (1.4%) |  |  |
| Agree | 4  (9.3%) | 49  (23.0%) | 12  (12.0%) | 6  (1.6%) |  |  |
| Strongly agree | 1  (2.3%) | 33  (15.5%) | 4  (4.0%) | 3  (0.8%) |  |  |
| No information provided | 32  (74.4%) | 81  (38.0%) | 62  (62.0%) | 347  (94.3%) |  |  |
| **I used the AdAM software for all registered patients as needed. (E1.11)** |  |  |  |  | **<0.001** | **<0.001** |
| Strongly disagree | 1  (2.3%) | 13  (6.1%) | 5  (5.0%) | 4  (1.1%) |  |  |
| Disagree | 2  (4.7%) | 12  (5.6%) | 10  (10.0%) | 4  (1.1%) |  |  |
| Neither agree nor disagree | 3  (7.0%) | 23  (10.8%) | 5  (5.0%) | 2  (0.5%) |  |  |
| Agree | 4  (9.3%) | 57  (26.8%) | 10  (10.0%) | 6  (1.6%) |  |  |
| Strongly agree | 1  (2.3%) | 26  (12.2%) | 7  (7.0%) | 4  (1.1%) |  |  |
| No information provided | 32  (74.4%) | 82  (38.5%) | 63  (63.0%) | 348  (94.6%) |  |  |
| **Using the AdAM software has made me more aware of medication risks. (E1.12)** |  |  |  |  | **<0.001** | **<0.001** |
| Strongly disagree | 1  (2.3%) | 15  (7.0%) | 6  (6.0%) | 7  (1.9%) |  |  |
| Disagree | 1  (2.3%) | 24  (11.3%) | 9  (9.0%) | 4  (1.1%) |  |  |
| Neither agree nor disagree | 1  (2.3%) | 34  (16.0%) | 14  (14.0%) | 2  (0.5%) |  |  |
| Agree | 7  (16.3%) | 42  (19.7%) | 6  (6.0%) | 5  (1.4%) |  |  |
| Strongly agree | 1  (2.3%) | 17  (8.0%) | 3  (3.0%) | 3  (0.8%) |  |  |
| No information provided | 32  (74.4%) | 81  (38.0%) | 62  (62.0%) | 347  (94.3%) |  |  |
| **Using the AdAM software has given me new knowledge that I can apply to other patients. (E1.13)** |  |  |  |  | **<0.001** | **<0.001** |
| Strongly disagree | 1  (2.3%) | 12  (5.6%) | 7  (7.0%) | 6  (1.6%) |  |  |
| Disagree | 0  (0.0%) | 23  (10.8%) | 9  (9.0%) | 3  (0.8%) |  |  |
| Neither agree nor disagree | 5  (11.6%) | 35  (16.4%) | 9  (9.0%) | 4  (1.1%) |  |  |
| Agree | 5  (11.6%) | 43  (20.2%) | 11  (11.0%) | 4  (1.1%) |  |  |
| Strongly agree | 0  (0.0%) | 19  (8.9%) | 2  (2.0%) | 4  (1.1%) |  |  |
| No information provided | 32  (74.4%) | 81  (38.0%) | 62  (62.0%) | 347  (94.3%) |  |  |
| **The communication about the project from KVWL and BARMER inspired me to implement AdAM in my medical practice. (E1.14)** |  |  |  |  | **<0.001** | 0.589 |
| Strongly disagree | 1  (2.3%) | 13  (6.1%) | 5  (5.0%) | 4  (1.1%) |  |  |
| Disagree | 1  (2.3%) | 20  (9.4%) | 10  (10.0%) | 3  (0.8%) |  |  |
| Neither agree nor disagree | 2  (4.7%) | 35  (16.4%) | 7  (7.0%) | 7  (1.9%) |  |  |
| Agree | 6  (14.0%) | 46  (21.6%) | 14  (14.0%) | 7  (1.9%) |  |  |
| Strongly agree | 1  (2.3%) | 18  (8.5%) | 2  (2.0%) | 2  (0.5%) |  |  |
| No information provided | 32  (74.4%) | 81  (38.0%) | 62  (62.0%) | 345  (93.8%) |  |  |
| **KVWL's involvement in the project motivated me to use AdAM. (E1.15)** |  |  |  |  | **<0.001** | 0.619 |
| Strongly disagree | 0  (0.0%) | 18  (8.5%) | 6  (6.0%) | 5  (1.4%) |  |  |
| Disagree | 1  (2.3%) | 28  (13.1%) | 10  (10.0%) | 4  (1.1%) |  |  |
| Neither agree nor disagree | 4  (9.3%) | 37  (17.4%) | 13  (13.0%) | 9  (2.4%) |  |  |
| Agree | 5  (11.6%) | 34  (16.0%) | 7  (7.0%) | 2  (0.5%) |  |  |
| Strongly agree | 1  (2.3%) | 14  (6.6%) | 2  (2.0%) | 3  (0.8%) |  |  |
| No information provided | 32  (74.4%) | 82  (38.5%) | 62  (62.0%) | 345  (93.8%) |  |  |

* In consultation with the authors of the German version of the Organisational Readiness for Implementing Change measure (ORIC), we omitted the last item because it performed very poorly in the pretest. Our ORIC therefore has one item less.

Table S3: Internal consistency of questionnaire-based measures and subscales assessed using Cronbach’s α coefficients.

| **Scale** | **No. of items** | **Cronbach’s α** |
| --- | --- | --- |
| PAR measure | 23 | 0.92 |
| ORIC measure* | 9 | 0.96 |
| ORIC (change commitment; item 1-5) | 5 | 0.92 |
| ORIC (change efficacy; item 6-9)* | 4 | 0.97 |
| G-NoMAD Total | 20 | 0.93 |
| G-NoMAD Subscale Coherence | 4 | 0.87 |
| G-NoMAD Subscale Cognitive Participation | 4 | 0.84 |
| G-NoMAD Subscale Collective Action | 7 | 0.70 |
| G-NoMAD Subscale Reflexive Monitoring | 5 | 0.85 |

* In consultation with the authors of the German version of the Organisational Readiness for Implementing Change measure (ORIC), we omitted the last item because it performed very poorly in the pretest. Our ORIC therefore has one item less.

Table S4: Sensitivity analyses of hierarchical clustering solutions using alternative distance measures and agglomeration methods.

| **Distance measure** | **Agglomeration method** | **Optimal no. of groups*** | **Silhouette index** | **Group distribution** |
| --- | --- | --- | --- | --- |
| Euclidean | Ward D | 3 | 0.265 | 1: 43, 2: 213, 3: 100 |
|  | Ward D2 | 3 | 0.233 | 1: 42, 2: 201, 3: 113 |
|  | Complete | 2 | 0.512 | 1: 353, 2: 3 |
|  | Average | 2 | 0.512 | 1: 353, 2:3 |
| Manhattan | Complete | 2 | 0.278 | 1: 126, 2: 230 |
|  | Average | 2 | 0.424 | 1: 353, 2:3 |

The table presents the optimal number of groups identified for each clustering specification based on the highest mean silhouette index, together with the corresponding group distributions. All clustering variables were standardised prior to analysis.


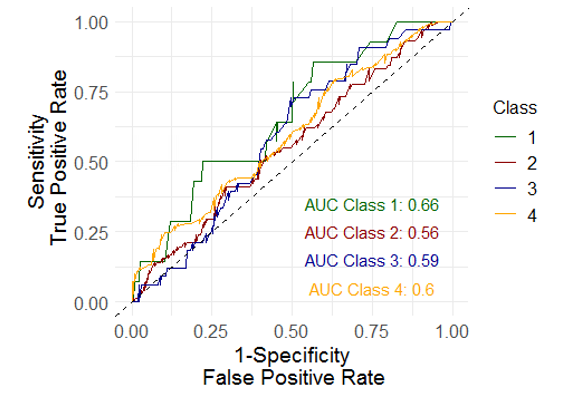


Figure S8: Receiver Operating Characteristic (ROC) curves and corresponding Area Under the Curve (AUC) values for the Random Forest (RF) model across four distinct adoption patterns. The RF model includes patient characteristics, practice profile information, and randomization wave.


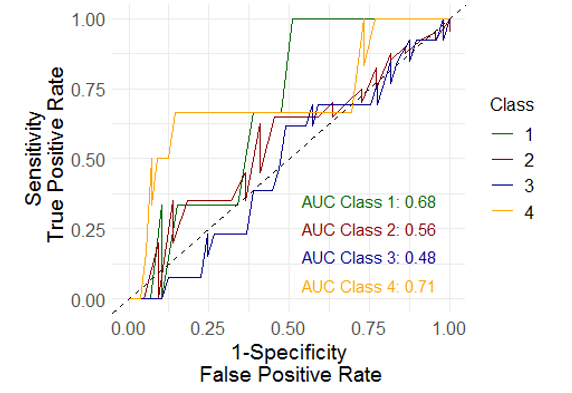


Figure S9: Receiver Operating Characteristic (ROC) curves and corresponding Area Under the Curve (AUC) values for the Random Forest (RF) model across four distinct adoption patterns. The RF model includes patient characteristics, practice profile information, randomization wave and the following survey questions: A3, A3, A7, A8, B1.1, B1.5, B2.1 – B2.10, B2.12-23, D1.1.1, and D1.1.2 (candidate explanatory variables that are completed by at least 29% of practices in all adoption patterns). The RF model does only consider data without missing values.


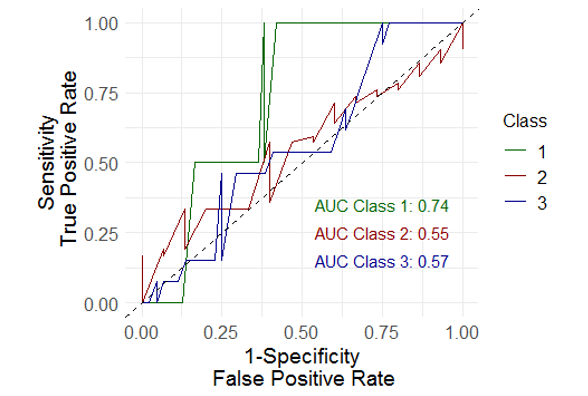


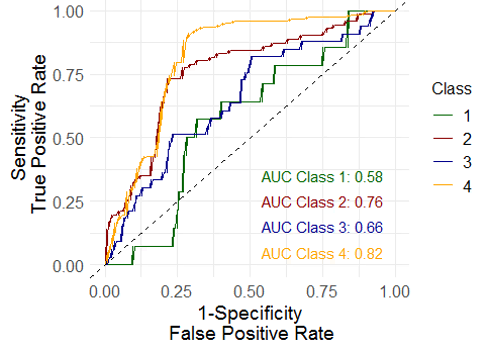
Figure S10: Receiver Operating Characteristic (ROC) curves and corresponding Area Under the Curve (AUC) values for the Random Forest (RF) model across three distinct adoption patterns. The RF model includes patient characteristics, practice profile information, randomization wave and the following survey questions: A3, A4, A7, A8, B2.8-23, D1.1.1-3, D1.2.1-5, D1.2.9, E1.1.1-3, E1.7-10, E1.12-15 (candidate explanatory variables that are completed by at least 49% of practices in Pattern 1-3). The RF model does only consider data without missing values.

Figure S11: Receiver Operating Characteristic (ROC) curves and corresponding Area Under the Curve (AUC) values for the Gradient Boosting model (GBM) across four distinct adoption patterns. The GBM model includes patient characteristics, practice profile information, randomisation wave, and an indicator that shows whether the GP participated in the survey.


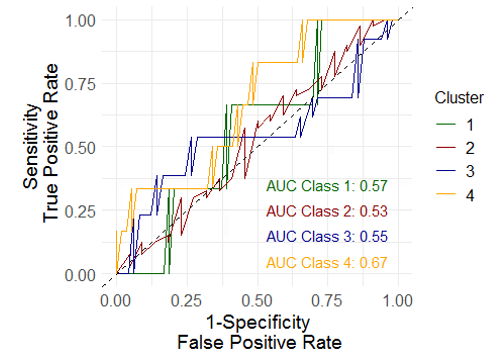

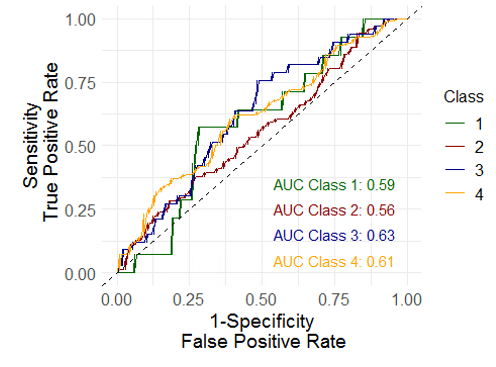
Figure S12: Receiver Operating Characteristic (ROC) curves and corresponding Area Under the Curve (AUC) values for the Gradient Boosting model (GBM) across four distinct adoption patterns. The GBM model includes patient characteristics, practice profile information, and randomization wave.

Figure S13: Receiver Operating Characteristic (ROC) curves and corresponding Area Under the Curve (AUC) values for the Gradient Boosting model (GBM) across four distinct adoption patterns. The GBM model includes patient characteristics, practice profile information, randomization wave and the following survey questions: A3, A3, A7, A8, B1.1, B1.5, B2.1 – B2.10, B2.12-23, D1.1.1, and D1.1.2 (candidate explanatory variables that are completed by at least 29% of practices in all adoption patterns). The RF model does only consider data without missing values.


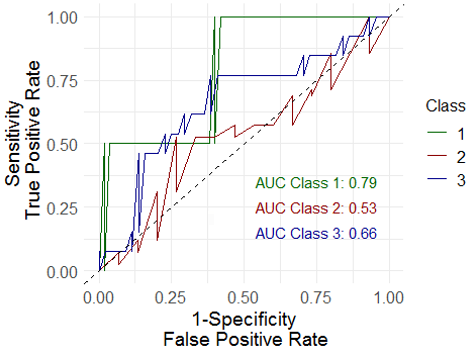


Figure S14: Receiver Operating Characteristic (ROC) curves and corresponding Area Under the Curve (AUC) values for the Gradient Boosting model (GBM) across three distinct adoption patterns. The GBM model includes patient characteristics, practice profile information, randomization wave and the following survey questions: A3, A4, A7, A8, B2.8-23, D1.1.1-3, D1.2.1-5, D1.2.9, E1.1.1-3, E1.7-10, E1.12-15 (candidate explanatory variables that are completed by at least 49% of practices in Pattern 1-3). The RF model does only consider data without missing values.
